# Nanoparticle-based therapeutic platforms for inflammatory disorders of the urinary system: Engineering local delivery, biointerface regulation, and smart responsive therapy

**DOI:** 10.1016/j.mtbio.2026.103673

**Published:** 2026-09-16

**Authors:** Wang Wang, Jiayi Ma, Changhao Hou, Jiasheng Chen, Kai Ni

**Affiliations:** Department of Urology, Shanghai Sixth People's Hospital Affiliated to Shanghai Jiao Tong University School of Medicine, Shanghai, 200233, China

**Keywords:** Inflammatory urinary disorders, Nanoparticle-based drug delivery, Targeted therapy, Theranostics, Precision medicine

## Abstract

Inflammatory diseases of the urinary system, including urinary tract infection (UTI), catheter-associated urinary tract infection (CAUTI), interstitial cystitis/bladder pain syndrome (IC/BPS), and chronic prostatitis/chronic pelvic pain syndrome (CP/CPPS), are associated with high recurrence, complex pathogenesis, and limited therapeutic durability. Conventional treatments are constrained by antimicrobial resistance, biofilm persistence, intracellular bacterial reservoirs, urine washout, urothelial and prostatic barriers, indwelling device surfaces, and heterogeneous neuroimmune inflammatory mechanisms. Nanoparticles provide versatile platforms to address these challenges through tunable physicochemical properties, controlled release, local retention, tissue penetration, and intrinsic material functions. This review summarizes the pathological basis and therapeutic limitations of major inflammatory urinary disorders and discusses recent advances in organic, inorganic, and hybrid nanoplatforms. In infectious diseases, nanoparticles can enhance antibiotic delivery, promote biofilm penetration and intracellular pathogen clearance, and functionalize catheter or stent surfaces with antibacterial, antibiofilm, and anti-encrustation properties. In noninfectious disorders, they support intravesical retention, glycosaminoglycan-layer repair, oxidative-stress modulation, immune regulation, pain-pathway intervention, and prostatic or rectal delivery. Emerging applications in upper UTI and renal inflammatory injury further expand their therapeutic scope. Finally, we discuss current translational barriers, including model fidelity, long-term safety, manufacturing control, clinical evidence, and rational functional integration.

## Highlights

• Translational framework maps nanoparticle functions in urinary inflammation.
• Disease-specific design tailors nanoparticle properties to clinical and pathological barriers.
• Biointerface regulation is emphasized through catheter surface coatings and mucosal retention systems.
• Stimuli-responsive nanoplatforms use pH, ROS, enzymes, or light for regulation.
• Translation challenges in safety, manufacturing, and endpoints are summarized.

## Graphical abstract

**Figure:**
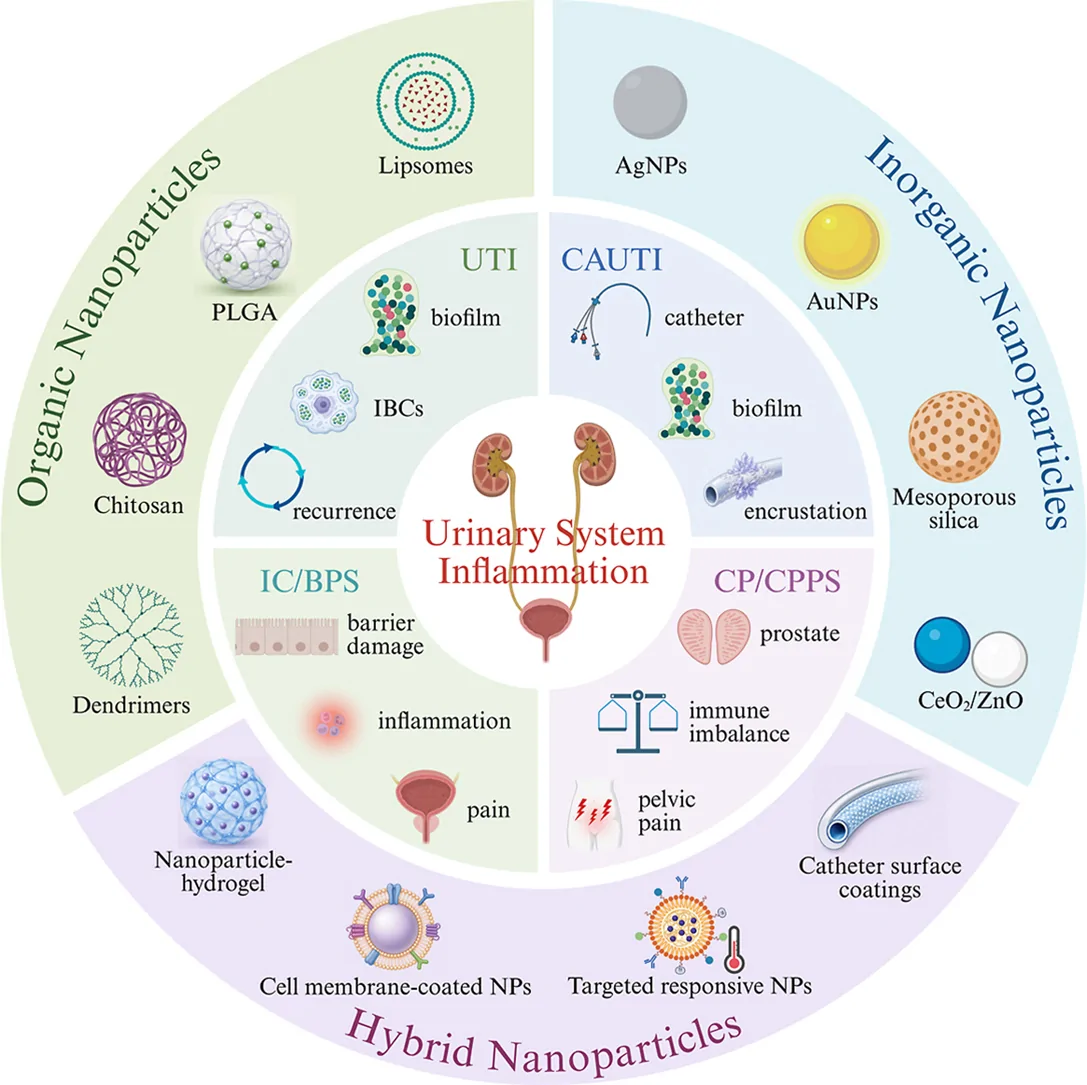

## 1 Introduction

Inflammatory diseases of the urinary system are major public health problems worldwide because of their high incidence, frequent recurrence, and therapeutic complexity [1,2]. They include infectious disorders caused by pathogenic microorganisms, particularly urinary tract infection (UTI) and catheter-associated urinary tract infection (CAUTI). UTIs affect approximately 150 million people worldwide each year, while about 75% of hospital-acquired UTIs are associated with urinary catheterization [3]. Inflammatory urinary disorders also include noninfectious conditions characterized by chronic inflammation, immune dysregulation, and neural sensitization, such as interstitial cystitis/bladder pain syndrome (IC/BPS) and chronic prostatitis/chronic pelvic pain syndrome (CP/CPPS). IC/BPS symptoms are estimated to affect approximately 2.7–6.5% of adult women in the United States, whereas CP/CPPS affects approximately 2–10% of men worldwide [4,5]. These disorders are often recurrent or persistent, substantially impair quality of life, and, in some patients, may lead to structural organ damage, functional impairment, or systemic complications.

Current management of inflammatory urinary disorders remains centered on conventional pharmacotherapy and symptomatic interventions. Antibiotics remain the mainstay of treatment for UTI and CAUTI [6,7], whereas IC/BPS and CP/CPPS are primarily managed with oral medications, intravesical instillation, physical therapy, neuromodulation, and multimodal care [8,9]. However, antimicrobial resistance, biofilm-associated persistence, and recurrent infection continue to challenge treatment [10,11]. In noninfectious disorders, complex multifactorial pathogenesis often limits conventional drugs to partial symptom relief, with inconsistent long-term efficacy and potential adverse effects [12]. Urine washout, cyclic bladder filling and emptying, urothelial barriers, limited prostatic penetration, and indwelling foreign surfaces further constrain local drug delivery and sustained activity [13,14].

Nanoparticles (NPs) are important platforms in drug delivery and biomaterials research and offer new perspectives for treating inflammatory urinary disorders [15]. By tuning particle size, surface charge, material composition, and surface ligands, they can enhance drug loading (DL), targeted delivery, and controlled release, thereby increasing local drug concentrations, prolonging exposure, and reducing systemic toxicity. Some inorganic and hybrid nanomaterials also have intrinsic antibacterial, antioxidant, photothermal, or imaging properties, expanding their potential in anti-infective therapy, inflammation modulation, and theranostics [[16], [17], [18]].

Guided by clinical needs, this review summarizes the pathogenesis and therapeutic limitations of UTI, CAUTI, IC/BPS, and CP/CPPS, introduces the major categories and functional characteristics of nanoparticles, reviews recent advances in the application of different nanoplatforms in these disorders, and discusses translational challenges and future directions (Fig. 1). This review aims to inform the development of urinary drug-delivery systems and precision therapeutic strategies.

**Fig. 1:**
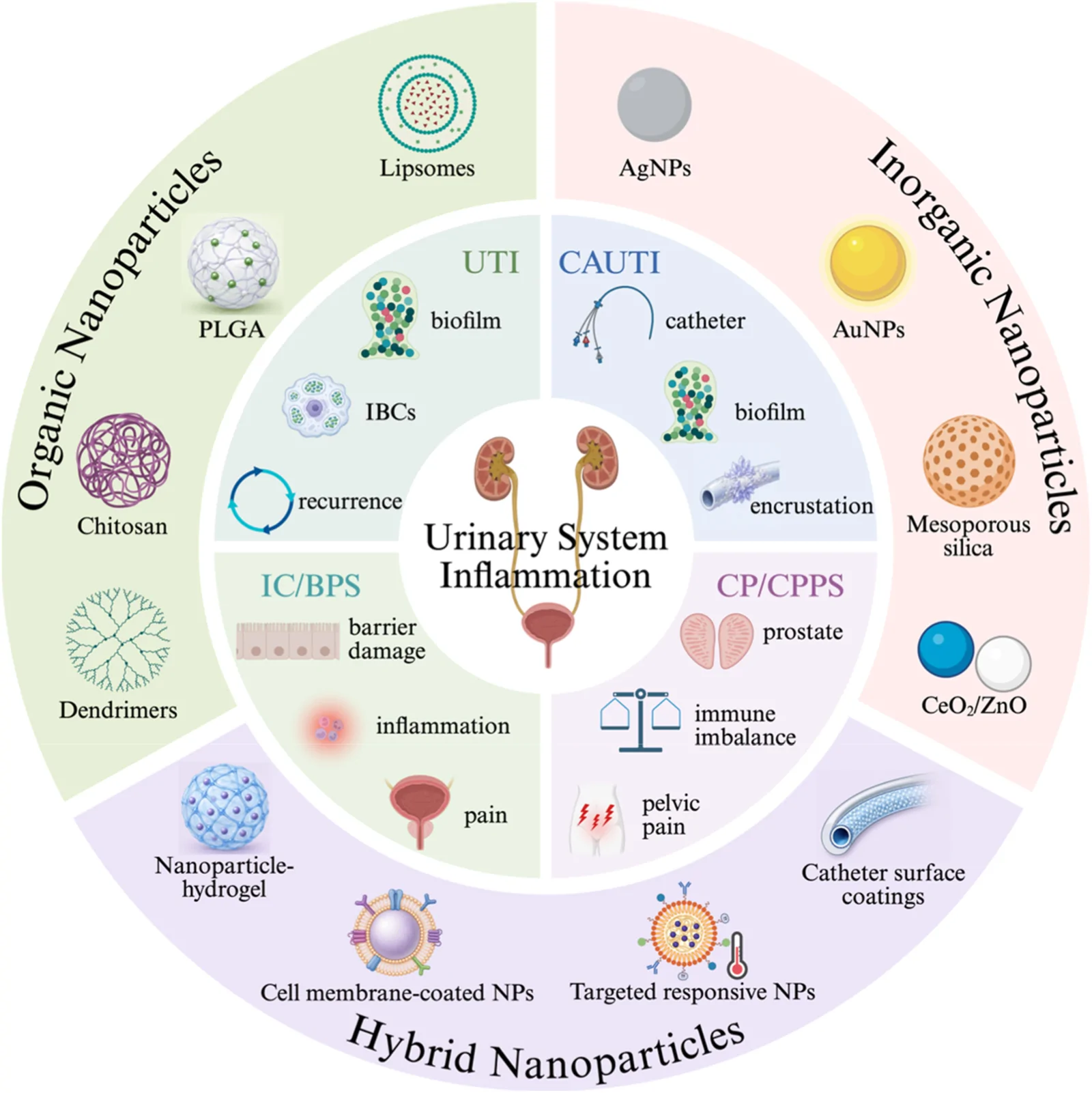
**Illustration of organic, inorganic, and hybrid nanoparticle-based platforms for the treatment of urinary system inflammation.** Created with BioRender.com. **Abbreviations:** IBCs, intracellular bacterial communities; PLGA, poly (lactic-co-glycolic acid).

## 2 Pathological basis of inflammatory diseases of the urinary system and limitations of conventional therapies

Inflammatory disorders of the urinary system can be broadly classified as infectious or noninfectious according to pathogenesis, with distinct pathological mechanisms and management strategies (Fig. 2).

**Fig. 2:**
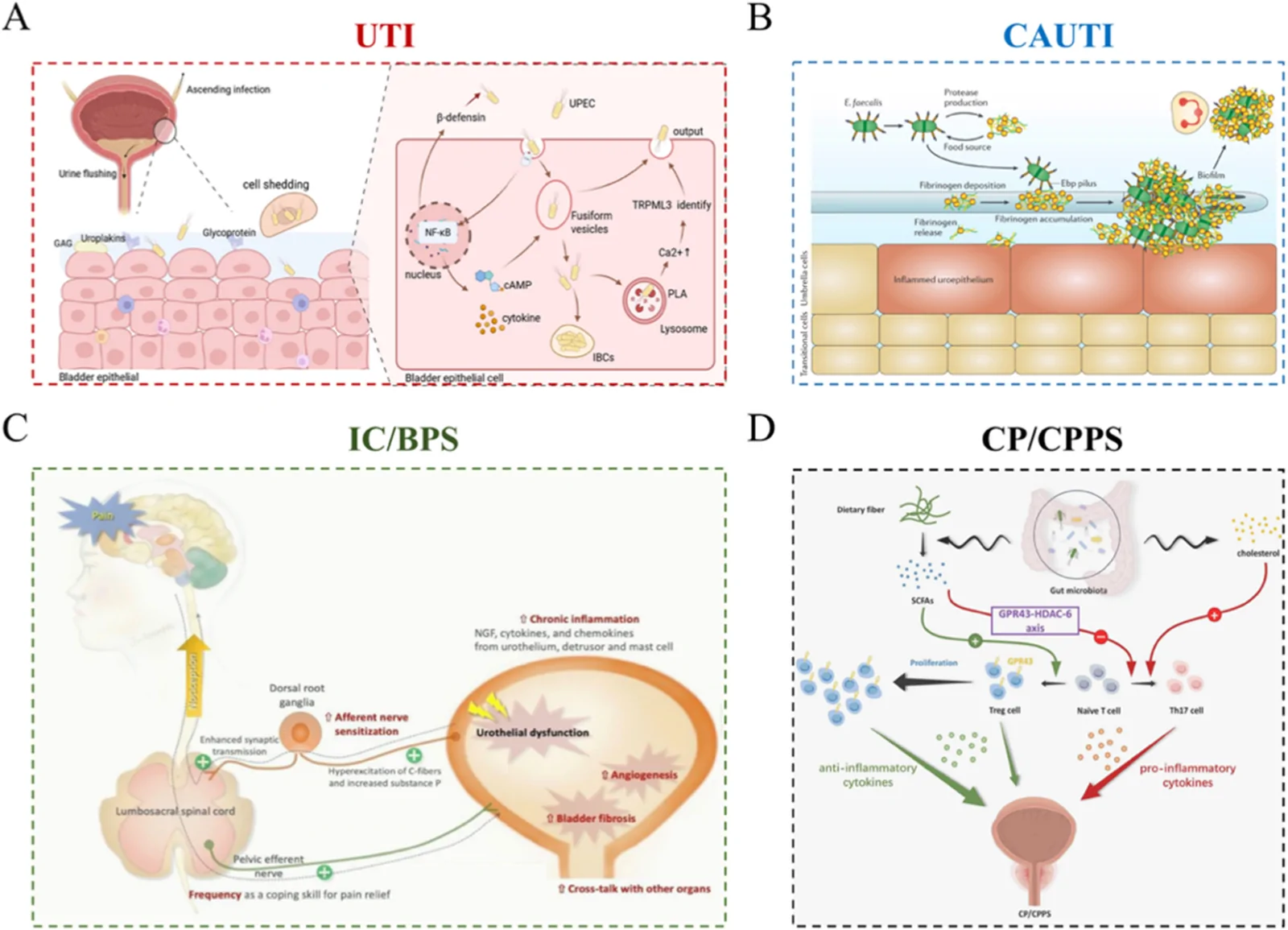
**Disease-specific pathological mechanisms and intervention targets in urinary system inflammation. A: Urinary Tract Infection (UTI).** UPEC adheres to and invades bladder epithelial cells, forming intracellular bacterial reservoirs that promote persistence and recurrence. Reproduced with permission from Hou et al. [19]. **B: Catheter-associated Urinary Tract Infection (CAUTI).** Catheterization induces urothelial inflammation and fibrinogen deposition on the catheter surface. *Enterococcus faecalis* exploits fibrinogen for adhesion and biofilm formation, supporting persistent catheter-associated infection. Reproduced with permission from Flores-Mireles et al. [20]. **C: Interstitial Cystitis/Bladder Pain Syndrome (IC/BPS).** Urothelial dysfunction, chronic inflammation, nerve sensitization, angiogenesis and bladder fibrosis jointly drive pain and urinary symptoms. Reproduced with permission from Hu et al. [21]. **D: Chronic Prostatitis/Chronic Pelvic Pain Syndrome (CP/CPPS).** Gut microbiota-derived metabolites regulate the Th17/Treg balance through the GPR43–HDAC6 axis, shaping local inflammatory responses. Reproduced with permission from Deng et al. [22].

### 2.1 Infectious inflammatory diseases of the urinary system

#### 2.1.1 Urinary tract infection (UTI)

UTI is one of the most common bacterial infectious diseases worldwide, affecting approximately 150 million individuals each year [6]. It can involve the urethra, bladder, ureters, and kidneys, with clinical manifestations ranging from asymptomatic bacteriuria and acute cystitis to pyelonephritis; in severe cases, it may progress to urosepsis [23,24]. Uropathogenic *Escherichia coli* (UPEC) is the predominant causative pathogen, followed by *Klebsiella pneumoniae*, *Staphylococcus saprophyticus*, *Enterococcus* spp., *Proteus* spp., and other organisms [25,26].

UTI persistence and recurrence depend largely on bacterial reservoirs that evade host clearance and antimicrobial therapy [27]. UPEC can adhere to the urothelium through adhesins such as FimH [28,29], subsequently invade epithelial cells, and form intracellular bacterial communities (IBCs) that promote immune evasion and antibiotic tolerance [30]. UPEC can also form extracellular biofilms, further increasing tolerance to antimicrobial agents and host defenses (Fig. 2A) [19,31,32].

Antibiotic therapy remains central to UTI management [33], with commonly used agents including amoxicillin, trimethoprim (TMP)/sulfamethoxazole, nitrofurantoin, and fluoroquinolones [1,34]. However, short-course oral or systemic therapy is poorly equipped to overcome urinary washout, mucosal barriers, biofilm protection, and intracellular bacterial persistence [10,35,36]. Urine dilution and voiding reduce exposure in the bladder lumen and at the urothelial surface, whereas biofilms and IBCs restrict penetration, allowing bacteria to persist and contribute to recurrence [37]. Prolonged or repeated antibiotic use may select for resistant organisms and disrupt the intestinal and urinary microbiota [38,39].

Accordingly, the key challenge in UTI treatment is no longer limited to the selection of antibacterial agents, but lies in improving local drug retention, mucosal and biofilm penetration, and intracellular pathogen clearance under conditions where urine washout, mucosal and biofilm barriers, and intracellular bacterial reservoirs coexist. Nanosystems with mucoadhesive, controlled-release, intracellular-delivery, and multimodal antibacterial properties therefore represent important complementary strategies to address these therapeutic bottlenecks.

#### 2.1.2 Catheter-associated urinary tract infection (CAUTI)

CAUTI is a major hospital-acquired UTI and is closely associated with long-term indwelling catheterization [20,[40], [41], [42]]. After catheter insertion, urinary constituents and host proteins rapidly deposit on the material surface, forming a conditioning film that promotes bacterial adhesion [43,44]. Pathogens then enter the urinary tract through intraluminal or extraluminal routes and progressively establish mature biofilms on the catheter surface [11,45,46].

Catheter-associated biofilms are a major reason why CAUTI is difficult to treat [47]. Their extracellular polymeric matrix limits antibiotic diffusion and creates a hypoxic, nutrient-depleted, metabolically quiescent microenvironment that reduces antimicrobial susceptibility [48,49]. Long-term catheterization is also frequently accompanied by encrustation, obstruction, mechanical irritation, and local inflammation, which further promote bacterial colonization and persistent infection (Fig. 2B) [20,50,51].

Current CAUTI prevention and management rely mainly on aseptic catheter insertion, minimization of catheter dwell time, use of closed drainage systems, catheter replacement when clinically indicated, and culture- and susceptibility-guided antimicrobial therapy for symptomatic CAUTI [2]. Although these measures can reduce infection risk or limit persistent bacterial sources, they do not directly interfere with conditioning-biofilm formation, early bacterial adhesion, or biofilm maturation on catheter surfaces [40,41]. Silver-alloy and antibiotic-coated catheters have been used in some healthcare settings [52,53]; however, currently available materials still face challenges such as poorly controlled release of active components, limited long-term antibacterial activity, insufficient eradication of mature biofilms, inconsistent anti-encrustation performance, and potential cytotoxicity [54,55].

Beyond standardized catheter care and antibiotic therapy, CAUTI prevention requires functional materials that act directly at the catheter surface to reduce early bacterial adhesion, inhibit biofilm maturation, maintain sustained local antibacterial activity, and preserve anti-encrustation performance and biocompatibility [56]. Nanocoatings, nanocomposite catheters, and nanoparticle-hydrogel composite coatings can integrate anti-adhesive, contact-killing, sustained-release, antibiofilm, and anti-encrustation functions, giving them considerable translational potential [57].

### 2.2 Noninfectious inflammatory diseases of the urinary system

#### 2.2.1 Interstitial cystitis/bladder pain syndrome (IC/BPS)

IC/BPS is a chronic disorder characterized primarily by bladder pain, urinary frequency, urgency, and nocturia [58,59]. It can substantially impair daily activities, psychosocial functioning, and quality of life, and imposes a considerable disease burden [60,61]. The etiology and pathogenesis of IC/BPS have not been fully elucidated. Available evidence indicates that disease development is associated with urothelial barrier dysfunction [62], aberrant mast-cell activation [63,64], neurogenic inflammation and central sensitization [65], autoimmunity [66,67], and genetic susceptibility [68]. These processes interact with one another, resulting in substantial interpatient heterogeneity in symptoms, underlying pathology, and treatment responses (Fig. 2C) [21].

The management of IC/BPS generally follows a stepwise approach, with lifestyle modification and behavioral interventions [[69], [70], [71], [72]], oral pharmacotherapy [73,74], intravesical instillation [75,76], neuromodulation [77], and surgery [78,79] introduced according to symptom severity, patient phenotype, and prior treatment response. Nevertheless, current treatment remains largely focused on symptom control and empiric combination regimens, and precise interventions capable of addressing distinct pathological subtypes are still lacking.

The major therapeutic challenges in IC/BPS arise from the marked heterogeneity of the disorder and the complexity of its local pathological environment. On the one hand, robust predictive biomarkers of treatment response are lacking, and the dominant pathological drivers—including urothelial barrier injury, mast-cell activation, immune abnormalities, oxidative stress, and neural sensitization—vary among patients, complicating patient stratification and therapeutic selection [67]. On the other hand, although intravesical administration has the anatomical advantage of acting directly on diseased mucosa, conventional instillations are diluted and eliminated by urine, have limited mucosal adhesion and tissue penetration [76].

Given these characteristics, nanodelivery systems combining mucoadhesion, controlled release, barrier repair, anti-inflammatory and antioxidant activity, and multitarget regulation are well suited to coexisting chronic inflammation and barrier injury in IC/BPS. Liposomes, chitosan, poly(lactic-co-glycolic acid) (PLGA) nanoparticles, glycosaminoglycan (GAG) self-assembled nanostructures, and nanoparticle-hydrogel composites may overcome limitations of conventional intravesical instillation by prolonging retention, enhancing mucosal interactions, enabling sustained release, and integrating multiple therapeutic functions.

#### 2.2.2 Chronic prostatitis/chronic pelvic pain syndrome (CP/CPPS)

Prostatitis is a common urological disorder in adult men, affecting approximately 9.3% of men during their lifetime [9]. The National Institutes of Health (NIH) classifies prostatitis into four categories, of which CP/CPPS is classified as category III [80]. It is characterized by persistent pelvic pain, urinary dysfunction, and impaired quality of life [81,82]. Unlike bacterial prostatitis, CP/CPPS usually lacks evidence of persistent infection and is associated with local immune dysregulation, chronic inflammation, oxidative stress, pelvic-floor dysfunction, peripheral and central neural sensitization, and psychosocial factors [83]. CP/CPPS is highly heterogeneous: some patients predominantly exhibit local inflammation and immune abnormalities, whereas others present chiefly with urinary symptoms, pelvic-floor muscle tenderness, neural sensitization, or psychosocial problems [84,85]. Accordingly, the UPOINT (Urinary, Psychosocial, Organ-specific, Infection, Neurologic/systemic, and Tenderness) phenotyping system has been used for patient stratification and multimodal treatment decision-making [80,86,87].

Treatment options for CP/CPPS include antibiotics, α-blockers, anti-inflammatory and analgesic agents, immunomodulators, phytotherapeutic preparations, and neuromodulators [82,83], as well as pelvic-floor physical therapy, psychological intervention, extracorporeal shock-wave therapy, and local thermotherapy [88,89]. However, most regimens remain empiric and symptom-directed, and no specific therapy has yet been shown to directly reverse the chronic neuroimmune inflammatory network. Antibiotics are appropriate only when infection is documented or strongly suspected, whereas long-term empiric use may increase the risk of resistance, dysbiosis, and adverse effects. α-Blockers and analgesics can alleviate selected symptoms or pain but have limited effects on persistent immune-mediated inflammation, oxidative stress, and tissue remodeling. Pelvic-floor rehabilitation and psychological interventions may improve some symptoms but do not resolve inadequate drug exposure within the prostate (Fig. 2D) [22,90].

The therapeutic bottleneck in CP/CPPS is not merely the limited range of available drugs; rather, existing agents often fail to achieve adequate exposure in inflamed prostatic tissue while also addressing interconnected inflammation, immune imbalance, and pain sensitization. The prostatic capsule, epithelial tight junctions, and local microcirculation restrict penetration into the prostatic interstitium and acini, and chronic pain sensitization may limit the benefit of local anti-inflammatory treatment alone.

Nanodelivery systems can enhance local drug accumulation and sustained release in the prostate and surrounding inflammatory tissues through size optimization, surface-ligand modification, responsiveness to the inflammatory microenvironment, and local administration. They may also integrate anti-inflammatory, antioxidant, immunomodulatory, and pain-modulating interventions, supporting a shift from empiric symptom control toward mechanism-guided multitarget therapy.

## 3 Characteristics of nanoparticles and their advantages in the treatment of inflammatory diseases of the urinary system

Nanoparticles generally refer to nanoscale particles or carrier systems used in drug delivery, diagnosis and theranostics [[91], [92], [93]]. Compared with conventional small-molecule drugs or topical formulations, nanoplatforms offer benefits beyond increased DL. By modulating material composition, particle size, surface charge, morphology, and surface functional groups, they can alter drug distribution, local retention, tissue penetration, and release profiles [94,95]. They can carry antibiotics, anti-inflammatory agents, natural compounds, proteins, peptides, and nucleic acids through encapsulation, adsorption, hydrophobic interactions, coordination, or conjugation, thereby improving drug stability, local exposure, and barrier penetration while reducing nonspecific toxicity [96].

Based on material composition and structural characteristics, nanoparticle platforms relevant to inflammatory urinary disorders can be broadly classified as organic, inorganic, and hybrid nanoparticles. Organic nanoparticles include liposomes, polymeric nanoparticles, dendrimers, and chitosan nanoparticles, and generally offer favorable biocompatibility, biodegradability, and flexibility in DL [97,98]. Inorganic nanoparticles include metallic nanoparticles such as silver, gold, and copper; metal oxide nanoparticles such as zinc oxide, copper oxide, and iron oxide; and nonmetallic inorganic nanomaterials such as mesoporous silica. Compared with organic carriers, inorganic nanoparticles typically exhibit greater structural stability and intrinsic antibacterial, antioxidant, photothermal, magnetically responsive, or imaging properties [99]. Hybrid nanoplatforms integrate the advantages of different materials through organic–inorganic composites, lipid–inorganic core–shell structures, polymer-coated metallic cores, cell-membrane cloaking, or incorporation into local delivery matrices, thereby improving drug-loading capacity, structural stability, local retention, and multifunctional synergy [100]. These three classes differ in drug-loading mechanisms, biocompatibility, intrinsic material functions, local residence capacity, and translational challenges; the choice of platform should be guided by the disease site, dominant pathological barriers, route of administration, and safety requirements (Fig. 3) [101].

**Fig. 3:**
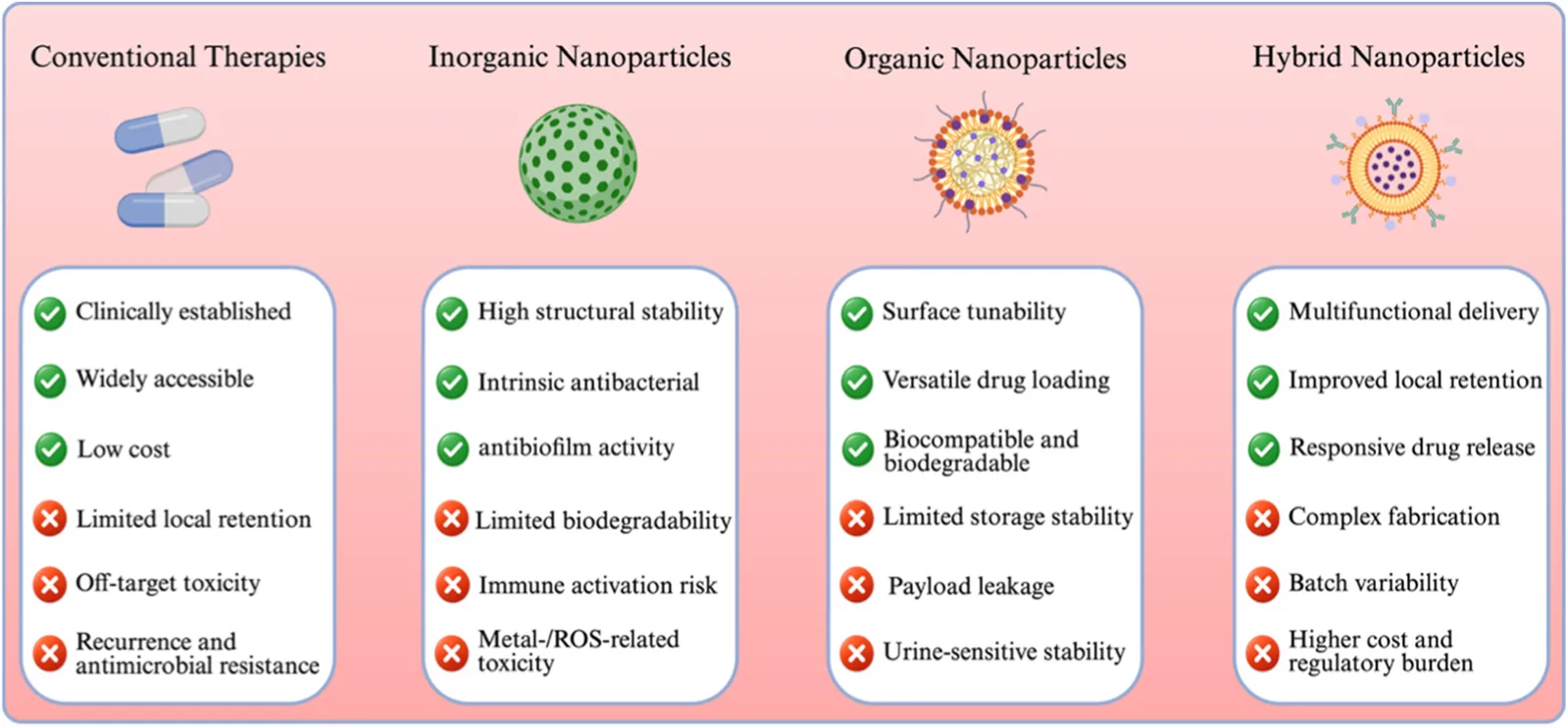
**Advantages and disadvantages of conventional therapies, inorganic nanoparticles, organic nanoparticles, and hybrid nanoparticles.** Created with BioRender.com.

The rationale for introducing nanoparticles into the treatment of inflammatory urinary disorders arises largely from the unique anatomical, physiological, and pathological features of the urinary system [102]. Although the etiologies of individual diseases differ, they share barriers that limit conventional drug delivery and sustained activity. In UTI, the main challenges include pathogen adhesion to and invasion of the urothelium, intracellular persistence, and the protection afforded by biofilms against antibiotics and host immune responses. CAUTI is further influenced by conditioning-biofilm formation, early bacterial colonization, biofilm maturation, and encrustation at the catheter surface, reflecting persistent infection at the catheter–urine–microbe interface. By contrast, IC/BPS and CP/CPPS usually lack a single, clearly defined pathogenic driver, and their course involves urothelial barrier injury, amplification of chronic inflammation, oxidative stress, immune imbalance, neural sensitization, and tissue remodeling. Meanwhile, continuous urine washout, cyclic bladder filling and emptying, the urothelial barrier, the surfaces of indwelling devices such as catheters, and prostatic capsular and epithelial barriers can all limit the retention, diffusion, and sustained action of free drugs at disease sites [103]. Accordingly, the key to treating inflammatory urinary disorders is not simply increasing the administered dose, but improving local delivery routes, lesion accessibility, and the duration of effective exposure in specific disease settings.

Within this framework, the three classes of nanoplatforms serve distinct functional roles. Organic nanosystems are generally well suited to drug protection, mucoadhesion, local controlled release, cellular uptake, and nucleic acid or protein delivery, making them highly compatible with intravesical instillation, intracellular pathogen clearance, urothelial barrier repair, and local prostatic delivery [98]. Inorganic nanomaterials are more appropriate for settings in which the material itself must exert an effect, including drug-resistant infections, mature biofilms, and catheter-surface colonization. They can provide antibacterial or antibiofilm activity that does not rely exclusively on traditional antibiotics through metal-ion release, reactive oxygen species (ROS) modulation, membrane damage, enzyme-like catalysis, or photothermal conversion [104]. Hybrid nanoplatforms enable the coordinated integration of drug delivery, therapeutic activity, and local retention or surface immobilization, and are therefore better suited to complex pathological processes or dynamic local environments [100]. For example, in CAUTI, hybrid systems can integrate antifouling, anti-adhesive, contact-killing, antibiofilm, anti-encrustation, and long-acting drug-release functions on catheter surfaces. In recurrent UTI, they may combine mucoadhesion, cellular uptake, and controlled antibiotic release to improve clearance of intracellular bacterial reservoirs. In IC/BPS and CP/CPPS, integration with hydrogels, cell membranes, or inflammation-responsive structures can enhance local retention and enable multitarget intervention. Accordingly, organic systems require particular attention to stability in urine and premature drug leakage; inorganic systems require careful assessment of local tissue compatibility, metal-ion release, and long-term metabolic clearance; and hybrid systems face more complex challenges related to manufacturing scale-up, batch consistency, and interactions among components.

Overall, nanoparticles should not be regarded simply as nanoscale reformulations of conventional drugs, but as tools for reconfiguring local therapy. In infectious inflammation, they can enhance drug stability, biofilm penetration, and intracellular delivery while reducing bacterial adhesion and maintaining local antibacterial activity. In noninfectious chronic inflammation, they can prolong drug exposure in the bladder or prostate and support barrier repair, inflammation suppression, oxidative-stress modulation, immune remodeling, and pain-related interventions. Based on the alignment between disease needs and material functions, the following sections discuss the roles and advantages of nanoparticles in urinary system inflammation in terms of biofilm and intracellular pathogen clearance, improved drug stability and local exposure, local retention and mucoadhesion, multifunctional synergistic therapy, and stimuli-responsive release (Fig. 4).

**Fig. 4:**
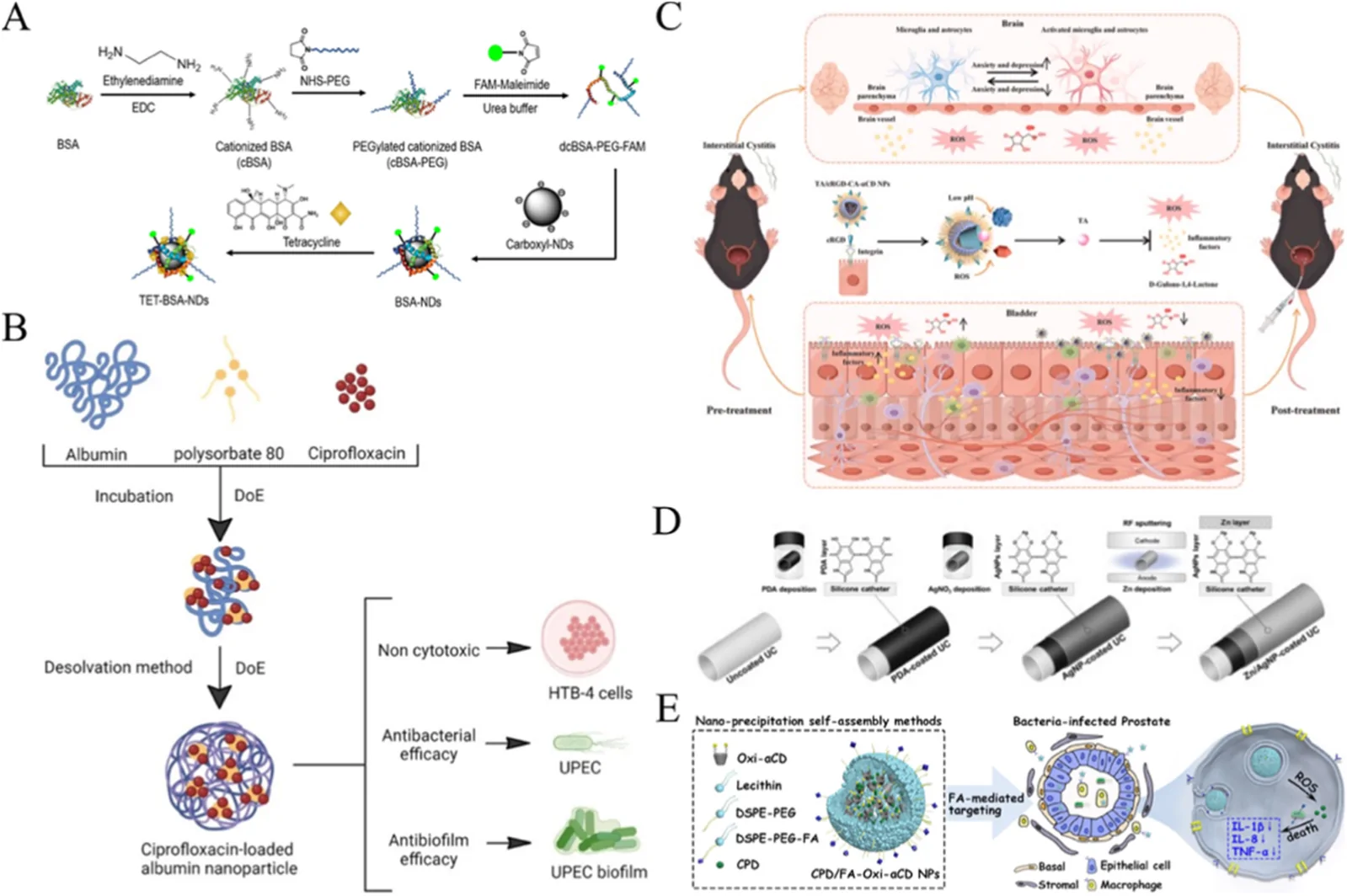
**Mechanism-oriented advantages of nanoparticle-based therapeutic strategies for urinary system inflammation. A: Biofilm and intracellular pathogen clearance.** BSA/PEG-functionalized nanodiamonds enhance TET dispersibility and intracellular delivery, promoting clearance of intracellular UPEC reservoirs. Reproduced with permission from Law et al. [105]. **B: Improved drug stability and effective local exposure.** CPF-loaded albumin nanoparticles improve CPF encapsulation, sustained release, antibacterial activity, and biofilm inhibition against UPEC. Reproduced with permission from Sánchez et al. [106]. **C: Local retention, mucoadhesion, and tissue penetration.** cRGD-functionalized TA-loaded nanoparticles enhance bladder retention, tissue penetration, and pH/ROS-responsive anti-inflammatory delivery. Reproduced with permission from Liu et al. [107]. **D: Multifunctional synergistic interface therapy.** A dual-layer AgNP/Zn nanoengineered catheter regulates Ag^+^ release, provides Zn^2+^-mediated antibacterial activity, and reduces adhesion, biofilm formation, and encrustation. Reproduced with permission from Won et al. [108]. **E: Stimuli-responsive targeted release.** Folate-modified CPD-loaded Oxi-αCD nanoparticles target inflamed prostatic tissue and enable ROS-responsive antibiotic release for chronic bacterial prostatitis. Reproduced with permission from Zheng et al. [109]. **Abbreviations**: BSA, bovine serum albumin; CPD, cefpodoxime proxetil; CPF, ciprofloxacin; cRGD, cyclic Arg-Gly-Asp; FA, folic acid; Oxi-αCD, oxidation-responsive α-cyclodextrin; PEG, polyethylene glycol; ROS, reactive oxygen species; TA, triamcinolone acetonide; TET, tetracycline.

### 3.1 Targeting biofilms, intracellular bacteria, and antimicrobial resistance

Biofilm formation contributes to persistent and recurrent UTI and CAUTI, whereas intracellular bacterial reservoirs are particularly relevant to recurrent UTI [19]. Conventional antibiotics may show limited efficacy against mature biofilms because matrix-associated diffusion barriers, altered local microenvironments, and biofilm-associated tolerance reduce effective exposure [110]. Nanoparticles can enhance penetration of the extracellular polymeric substance (EPS) matrix through nanoscale dimensions, tunable surface charge, and surface functionalization, delivering higher antimicrobial concentrations to deep biofilm layers and low-metabolic bacteria [111,112]. Cationic chitosan nanoparticles can interact electrostatically with negatively charged urothelial mucus and bacterial surfaces, prolonging retention and improving penetration [113,114].

Wen et al. [115] developed rosemary-oil-loaded chitosan nanophytosomes that enhanced antibacterial and antibiofilm activity in a multidrug-resistant *E. coli* UTI model while reducing inflammation and tissue injury. Beyond biofilms, IBCs and quiescent reservoirs in bladder epithelial cells remain major obstacles in recurrent UTI. Law et al. [105] developed tetracycline (TET)-loaded bovine serum albumin (BSA)-coated nanodiamonds (TET-BSA-NDs) for intravesical delivery to UPEC-infected bladders. The system was taken up by urothelial cells, colocalized with intracellular UPEC, released TET in acidic endosomes, and enhanced intracellular bacterial clearance, reduced bladder inflammation, and decreased residual IBCs (Fig. 4A).

Certain nanoparticles also exert antibacterial activity through multiple targets and mechanisms, potentially reducing reliance on single-target antibiotics and mitigating resistance-associated treatment failure. Combining nanoparticles with antibiotics is therefore considered a strategy to delay or overcome bacterial resistance [98,116]. For example, liposomal loading can increase drug accumulation at infection sites, reduce systemic toxicity, and protect antibiotics from local enzymatic inactivation [117], which is relevant to enzyme-mediated resistance in pathogens such as Enterobacteriaceae [118]. Nacucchio et al. [119] prepared liposomes composed of phosphatidylcholine and cholesterol to encapsulate piperacillin, successfully protecting the antibiotic from hydrolysis by staphylococcal β-lactamase and thereby preserving its antibacterial activity.

### 3.2 Enhancing drug stability and effective local exposure

Many antibacterial and anti-inflammatory drugs, as well as natural bioactive compounds, have poor water solubility, chemical instability, short *in vivo* half-lives, or limited local exposure in inflammatory urinary disorders [17]. Nanocarriers can protect drugs by encapsulation, adsorption, coordination, or conjugation; reduce degradation in biological fluids; and improve the dispersibility and stability of poorly soluble drugs, thereby enhancing delivery to urinary disease sites [94]. Sánchez et al. [106] developed ciprofloxacin-loaded BSA nanoparticles (CPF-loaded BSA NPs) with high encapsulation efficiency, colloidal stability, and concentration-dependent release in artificial urine and PBS. Compared with free CPF, CPF-loaded BSA NPs retained anti-UPEC activity, reduced cytotoxicity toward HTB-4 bladder cells, and showed greater activity against mature biofilms (Fig. 4B).

Plant-derived bioactive compounds often show poor aqueous solubility, limited membrane permeability, chemical instability, rapid clearance, and degradation in the gastrointestinal tract or biological fluids [94]. Curcumin and quercetin, for example, show anti-inflammatory and antioxidant activity *in vitro* but have low bioavailability. Man et al. [120] developed a nalidixic acid-curcumin combination delivered by cerium oxide nanoparticles. The system improved dispersion stability and showed antibacterial and antibiofilm activity against urinary pathogens such as *E. coli* and *Enterococcus faecalis*, while the enzyme-like activity of cerium oxide may further mitigate infection-associated oxidative stress.

### 3.3 Enhancing local retention and mucoadhesion

Intravesical instillation is an important local treatment for chronic inflammatory urinary disorders such as IC/BPS [121], but it is limited by urine washout, cyclic bladder filling and emptying, and the urothelial mucus barrier [76,122,123]. By tuning particle size, surface charge, and targeting ligands, nanoparticles can strengthen interactions with urinary mucosa or lesions; when combined with stimuli-responsive release or imaging capabilities, they can support lesion accumulation, prolonged retention, controlled release, and theranostic integration [124,125].

For example, positively charged nanoparticles, such as chitosan nanoparticles, can bind to negatively charged urothelial mucosa or bacterial surfaces through electrostatic interactions, thereby extending local drug action and improving mucosal penetration [113,114]. In addition, nanoparticle modification with hyaluronic acid (HA), mucin-binding materials, or inflammation-targeting ligands can further strengthen specific interactions with mucosal receptors, mucus-layer components, or lesion cells, enabling more prolonged local retention [76]. Liu et al. [107] developed cRGD-functionalized, triamcinolone acetonide (TA)-loaded pH/ROS dual-responsive nanoparticles (TA/cRGD-CA-αCD NPs) and used intravesical administration to treat cyclophosphamide (CYP)-induced IC/BPS in mice. cRGD binds to αvβ3 integrin, which is highly expressed in inflamed bladder tissue, allowing the nanoparticles to persist in the bladder longer than free fluorescent probes and non-targeted nanoparticles and to penetrate from the superficial urothelium into the submucosa and muscular layer. The system also exhibited pH- and ROS-responsive drug release, indicating that inflammation-targeted modification can integrate local adhesion, deep tissue penetration, and controlled release within one delivery platform, thereby addressing the limited retention and tissue penetration of conventional intravesical instillations (Fig. 4C).

Nanoparticles can also be combined with local delivery matrices or medical-device coatings to enhance residence and sustained release in the urinary environment. Hydrogels, polydopamine (PDA) coatings, zwitterionic polymers, and mucoadhesive polysaccharides can act as local retention or sustained-release interfaces under urine washout, catheter friction, and bladder emptying. Al-Enizi et al. [126] developed a cellulose-gel-based copper nanoparticle-hydrogel composite with adhesion, swelling-mediated retention, and sustained release. Mala et al. [127] used Spirulina-mediated silver nanoparticles (AgNPs) together with antibiotics to modify catheter surfaces, inhibiting bacterial adhesion and biofilm formation. Thus, local-retention strategies should be designed according to specific settings, including intravesical instillation, catheter coatings, or local urethral administration.

### 3.4 Multifunctional nanoplatforms for synergistic therapy

Inflammatory urinary disorders are driven by interconnected processes, including pathogen colonization, biofilm formation, urothelial barrier injury, local inflammation, oxidative stress, immune imbalance, and aberrant tissue repair [6]. Multimodal strategies integrating antibacterial, anti-inflammatory, antioxidant, barrier-protective, tissue-reparative, or sustained-release functions are therefore valuable. Nanoparticles can achieve such integration through drug co-loading, surface functionalization, or materials with intrinsic activities [128].

Won et al. [108] developed a dual-layer nanoengineered catheter for CAUTI prevention. A layer of AgNPs was deposited on silicone catheters and covered with a porous zinc layer. The silver layer provided sustained antibacterial activity, whereas the zinc layer regulated Ag^+^ release, reduced burst release, and, through Zn^2+^ release and ROS generation, inhibited early adhesion and biofilm formation. *In vitro*, the system reduced *E. coli* and *Staphylococcus aureus* colonization. In a rabbit catheterization model, it reduced biofilm formation, encrustation, urethral inflammation, and epithelial injury compared with silver-only and uncoated catheters. This study illustrates how spatially separated functional layers can integrate anti-adhesion, sustained antibacterial activity, antibiofilm activity, anti-encrustation performance, and biocompatibility at a single device interface (Fig. 4D). Alshehri et al. [129] prepared a hydrogel-AgNPs hybrid using carboxymethyl cellulose and polyvinyl alcohol. The material combined hydrogel swelling and biocompatibility with broad-spectrum silver antibacterial activity and sustained Ag^+^ release against clinically relevant uropathogens, including *E. coli*, *K*. *pneumoniae*, *P. aeruginosa*, and *S*. *aureus*. Abd Elkodous et al. [130] prepared anisotropic high-purity zinc oxide nanoparticles with antibacterial, antibiofilm, and potential antioxidant activity against multidrug-resistant uropathogens.

### 3.5 Stimuli-responsive drug release

Inflammatory lesions may exhibit changes in pH, ROS levels, enzyme activity, and other biochemical cues that can be exploited for responsive drug delivery [131]. However, the magnitude and consistency of these stimuli vary across diseases and patients. For example, pH alterations in IC/BPS may be relatively modest and heterogeneous, potentially limiting the specificity of pH-responsive systems when used alone. Dual- or multi-stimuli-responsive nanocarriers can improve activation specificity by integrating two or more pathological cues, either simultaneously or sequentially. In urinary inflammatory disorders, combinations such as pH/ROS responsiveness may therefore be particularly relevant, as demonstrated by dual-responsive nanoplatforms developed for IC/BPS and CP/CPPS [107]. Nevertheless, additional responsive modules should provide sufficient improvement in release selectivity to justify the increased formulation complexity. These cues provide endogenous triggers for lesion-specific delivery. Stimuli-responsive nanocarriers are designed by introducing cleavable bonds, protonatable groups, or environment-sensitive components into polymer backbones, drug-carrier linkers, or surface coatings. They remain relatively stable under physiological conditions but undergo disassembly, changes in hydrophilic–hydrophobic balance, swelling, or pore opening in diseased microenvironments, enabling site-specific controlled release [[131], [132], [133], [134]]. pH-responsive systems exploit local pH differences when sufficiently pronounced [131]. Common designs incorporate acid-labile linkages, such as hydrazone, imine, acetal, or ketal bonds, into nanocarriers, or use polymers containing protonatable amine groups, such as chitosan or poly(β-amino ester), as structural matrices [135]. Under low-pH conditions, acid-labile bonds can hydrolyze, or protonation of amine groups can alter carrier charge and hydrophilicity, causing nanoparticle swelling, structural loosening, or accelerated drug diffusion [136]. At near-physiological pH, the carrier remains relatively stable, thereby reducing premature drug leakage at nonlesional sites [137]. In addition to pH responsiveness, ROS-responsive materials are particularly suitable for chronic inflammatory settings marked by pronounced oxidative stress. These systems commonly incorporate ROS-sensitive groups, including thioketal bonds, diselenide bonds, or aryl boronate ester groups, into the material framework. When ROS levels rise, these structures undergo oxidative cleavage or conversion, disrupting the original hydrophobic core or cross-linked network of the nanoparticle and ultimately promoting release of the encapsulated drug [133].

Song et al. [138] constructed an amphiphilic polymeric framework from PLGA-selenocystamine conjugates and poly(ethylene glycol) (PEG) tetra-acid, using diselenide bonds to confer ROS responsiveness and subsequently loading CPF to create an antibacterial nanodelivery system. In an infection-associated high-ROS environment, oxidation of the diselenide bonds altered polymer-chain structure and hydrophilic-hydrophobic balance, causing gradual nanoparticle disassembly and accelerated CPF release. This design coupled drug release to the degree of oxidative stress at the infectious lesion and produced more sustained antibacterial activity than free drug under simulated high-ROS conditions *in vitro* while maintaining favorable biocompatibility. For prostatitis treatment, Zheng et al. [109] further combined lesion targeting with ROS-responsive release by developing folate-modified, cefpodoxime proxetil (CPD)-loaded ROS-responsive nanoparticles (CPD/FA-Oxi-αCD NPs). The system used an α-cyclodextrin derivative containing aryl boronate ester oxidation-sensitive groups as the ROS-responsive backbone and formed a nanostructure through self-assembly with lecithin and DSPE-PEG. Folate-modified DSPE-PEG was introduced at the surface to target folate receptors expressed by activated macrophages and upregulated in inflamed prostatic tissue. After entering prostatitis lesions, ROS oxidized the aryl boronate ester groups, altered carrier structure, and promoted CPD release; this process also consumed part of the excess ROS, thereby alleviating local oxidative stress while delivering the antibiotic. *In vivo*, the system promoted drug entry into prostatic tissue and glandular lumina, reduced bacterial burden and the expression of TNF-α, IL-1β, and IL-8, and attenuated pelvic pain sensitization in mice with chronic bacterial prostatitis (Fig. 4E).

Thus, stimuli-responsive systems are intended to improve the spatial and temporal control of drug release rather than simply increase drug release at lesions. However, responsive linkages or carrier structures may also undergo unintended activation or gradual degradation during storage, systemic transport, or local administration, resulting in premature drug leakage and reduced targeting specificity. Future formulations should therefore define appropriate activation thresholds and evaluate storage stability, baseline drug leakage, release kinetics under physiological conditions, and responsiveness under clinically relevant ranges of pathological stimuli. Balancing response sensitivity with formulation stability will be essential for clinical translation. When appropriately matched to disease-relevant stimuli, this strategy may improve local antibacterial or anti-inflammatory exposure while reducing off-target drug release and toxicity.

## 4 Nanoparticles in infectious inflammatory diseases of the urinary system

### 4.1 Nanoparticles in UTI

UTI is one of the most active areas of nano-enabled antibacterial and drug-delivery research. Studies have progressed from improving antibiotic delivery to addressing drug-resistant pathogens, biofilm penetration, and combined anti-infective and anti-inflammatory treatment (Fig. 5).

**Fig. 5:**
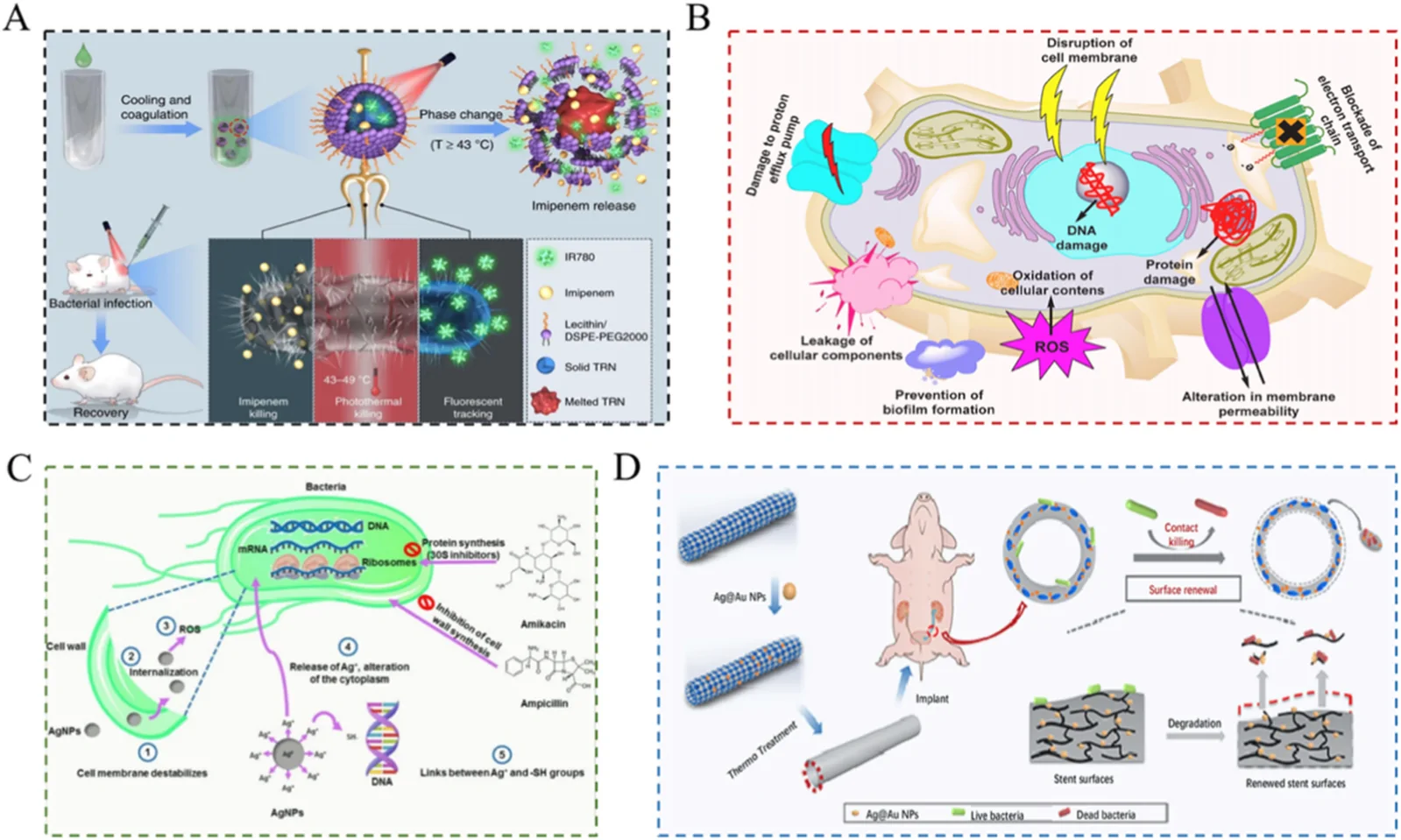
**Nanoparticle-enabled antimicrobial strategies for UTI. A:** A TRN co-loaded with IR780 and imipenem integrates fluorescence tracking, NIR-triggered photothermal killing, and antibiotic release. Reproduced with permission from Qing et al. [139]. **B:** Metallic nanoparticles exert antibacterial effects through membrane disruption, oxidative damage, biomolecular injury, and biofilm inhibition. Reproduced with permission from Qindeel et al. [140]. **C:** AgNPs combined with AMK and AMP enhance antibacterial activity against multidrug-resistant uropathogens through Ag^+^ release, ROS generation, membrane damage, and interference with bacterial DNA and proteins. Reproduced with permission from Lopez-Carrizales et al. [141]. **D:** Ag@Au nanoparticle-embedded biodegradable ureteral stents provide contact killing, surface renewal, biofilm suppression, and reduced stent-associated UTI risk. Reproduced with permission from Gao et al. [142]. **Abbreviations:** AMK, amikacin; AMP, ampicillin; NIR, near-infrared; TRN, thermo-responsive nanotransporter.

#### 4.1.1 Organic nanoparticles: Antibiotic delivery and intracellular clearance

Formulating conventional antibiotics as nanomedicines is a direct and potentially translatable strategy in nano-enabled UTI therapy. Organic nanocarriers offer biocompatibility, biodegradability, and flexible DL, and can be tailored for sustained release, mucoadhesion, cellular uptake, or reduced toxicity. Table 1 summarizes representative organic platforms, including particle characteristics, payloads, model systems, and outcomes. In UTI, these systems facilitate delivery to the urothelial surface, deep biofilm layers, and concealed intracellular pathogen reservoirs.

**Table 1:** Representative organic nanoparticle-based therapeutic platforms for UTI.

Nanoplatform	Particle characteristics	Active agent/Payload	Cell lines	Stage	Outcomes	Ref.
NSLC	Size: 176.70 ± 12.30 nm; PDI: 0.45 ± 0.01; ζ: −32.71 ± 2.07 mV	Rosemary oil, EE: ∼93.5%	—	*in vivo*	Enhanced antibacterial and antibiofilm activity, accompanied by renal protection.	115
LGseseTAPEG	Size: 153 nm (blank), 238 nm (DL 6.4%), 318.4 nm (DL 10.1%); PDI: 0.065 (blank), 0.125 (DL 6.4%), 0.158 (DL 10.1%)	CPF, DL: ∼6.4%/10.1%, EE: ∼68.4%/56.1%	L929, CCD986sk	*in vitro*	ROS-responsive CPF release enhanced antibacterial activity while preserving mammalian-cell cytocompatibility.	138
PLGA 503H-TMP-WGA/PLGA 2300H-TMP-WGA	Size: 190 ∼ 240 nm (503H), 280 ∼ 390 nm (2300H); PDI: 0.089 ∼ 0.154 (503H), 0.173 ∼ 0.208 (2300H); ζ: −55 ∼ −58 mV (503H), −55 ∼ −58 mV (2300H)	TMP, DL: 1.52% ∼ 19.48%	SV-HUCs	*in vitro*	Improved urothelial adhesion and supported intravesical TMP delivery.	143
CHX-NPs	Size: 198.8 nm	CHX, DL: 5.6%, EE: 45%	—	*in vitro*	Enabled tunable CHX release and prolonged inhibition of bacterial adhesion and colonization.	144
IMP/IR780@TRN	Size: 63.39 ± 7.75 nm; PDI: 0.27 ± 0.05	Imipenem, DL: 7.20%, EE: 74.9%	3T3, HUVECs	*in vivo*	Enabled NIR-triggered antibacterial therapy against sensitive and MDR bacteria at reduced antibiotic doses.	139
CPF-loaded BSA NPs	Size: 123 ± 3 nm; PDI: 0.13 ± 0.03; ζ: −31 ± 2 mV	CPF, EE: 87 ± 3%	HTB-4	*in vitro*	Improved CPF solubility and sustained exposure, enhancing antibacterial and antibiofilm performance.	106
FimHDG-Ferritin mRNA-LNP	—	—	293T, PBMCs, SV-HUC-1	—	Induced anti-FimH antibodies and FimH-specific T-cell responses, blocking bacterial adhesion.	145
GM-Nio	Size: 211.10 ± 3.50 nm; PDI: 0.20 ± 0.01	GM, EE: ∼10%	T24	*in vitro*	Enhanced intracellular GM delivery to bladder epithelial cells, reducing internalized UPEC burden.	146
CPF-loaded BSA NPs	Size: 238.2 nm	CPF HCl, EE: 38.11%	—	*in vitro*	Provided stable sustained CPF delivery with enhanced activity against common urinary pathogens.	147

**Abbreviations**: BSA, bovine serum albumin; CHX, chlorhexidine; CPF, ciprofloxacin; DL, drug loading; EE, encapsulation efficiency; GM, gentamicin; PBMCs, human peripheral blood mononuclear cells; HUVECs, human umbilical vein endothelial cells; IMP, imipenem; LNP, lipid nanoparticle; MDR, multidrug-resistant; NIR, near-infrared; Nio, niosome; PDI, polydispersity index; PLGA, poly(lactic-co-glycolic acid); ROS, reactive oxygen species; TMP, trimethoprim; TRN, thermo-responsive nanotransporter; WGA, wheat germ agglutinin; ζ, zeta potential.

Owing to favorable biodegradability, biocompatibility, and controlled release, PLGA nanoparticles are commonly used to load antibacterial agents such as CPF, ampicillin (AMP), gentamicin (GM), and nitrofurantoin [148]. Compared with free drugs, antibiotic-loaded PLGA nanoparticles can increase local accumulation and retention, prolong antibacterial activity, and may reduce bacterial tolerance [149]. Brauner et al. [150] prepared TMP-loaded microspheres and nanospheres using PLGA grades of different molecular weights for UTI therapy. Low-molecular-weight PLGA nanospheres achieved the highest DL, up to 29%, released drug within 24 h, and showed favorable 4 °C storage stability without lyophilization.

Other biodegradable polymeric nanosystems have also been used for UTI drug delivery. Phuengkham et al. [144] prepared chlorhexidine (CHX)-loaded PCL nanospheres by high-pressure emulsification and solvent evaporation, achieving sustained release and prolonged antibacterial activity compared with a physical mixture. Qing et al. [139] developed TRIDENT, a near-infrared (NIR)-responsive trifunctional thermosensitive nanotransporter co-encapsulating an antibiotic and a photosensitizer, thereby integrating fluorescence tracking, photothermal killing, and antibiotic-mediated antibacterial activity for multidrug-resistant infections (Fig. 5A).

Overall, organic nanocarriers in UTI are positioned primarily as delivery-optimization platforms. PLGA, PCL, liposomes and chitosan nanoparticles can serve distinct functions according to drug properties and therapeutic settings: biodegradable polymers are suited to sustained and stable antibiotic delivery; lipid- and protein-based carriers can improve cellular uptake and drug protection; and cationic or mucoadhesive materials are particularly suitable for local intravesical delivery and mucosal retention [91,151]. Their further translation will depend on continued optimization of drug-loading efficiency, stability in urine, intravesical residence time, and urothelial safety.

#### 4.1.2 Inorganic nanoparticles: Broad-spectrum antibacterial activity and antibiofilm effects

The advantages of inorganic nanomaterials in UTI therapy derive mainly from intrinsic material functions. Silver, zinc oxide, and mesoporous silica can exert bactericidal activity through metal-ion release, ROS generation, membrane disruption, protein-function interference, and DNA damage (Fig. 5B) [140]. They show inhibitory potential against multidrug-resistant bacteria and biofilm-associated infections and can be used alone or with antibiotics. Table 2 summarizes representative inorganic platforms, including particle features, active components, models, and antibacterial outcomes.

**Table 2:** Representative inorganic nanoparticle-based therapeutic platforms for UTI.

Nanoplatform	Particle characteristics	Active agent/Payload	Cell lines	Stage	Outcomes	Ref.
AgNPs	Size: 2 ∼ 20 nm	AgNPs	—	*in vitro*	Disrupted UPEC biofilms through membrane injury and ROS-mediated damage.	152
ZnO NPs	Size: 68.2 nm; ζ: −20.4 mV	ZnO	EAC	*in vitro*	Produced dose-dependent antibacterial and antibiofilm effects against UTI pathogens.	130
AgNPs	—	AgNPs; plant-derived capping/reducing components	MCF-7, VERO	*in vitro*	Showed broad antimicrobial and antioxidant activities exceeding those of the plant extract.	153
AgNPs/TiO_2_ NPs	—	AgNPs and TiO_2_ NPs	—	*in vitro*	Suppressed *Proteus* swarming and virulence through membrane/oxidative damage and fliL downregulation.	154
CuO NPs	Size: 15 ∼ 25 nm	CuO/Cu^2+^	RBCs	*in vitro*	Exhibited activity against CPF-resistant, ESBL-producing uropathogens through membrane and oxidative damage.	155
SNPs	Size: 75 ∼ 90 nm; ζ: −9.24 mV	SNPs combined with antibiotics	—	*in vitro*	Potentiated selected antibiotics against MDR urinary isolates through membrane permeabilization and oxidative stress.	156
ZnO NPs	Size: 20 ∼ 40 nm	ZnO	—	*in vitro*	Reduced *Candida* adhesion and biofilm-associated gene expression by downregulating ALS1/ALS3.	157
AgB NPs	Size: 85.25 nm	AgB NPs	—	*in vitro*	Inhibited UPEC growth and biofilm formation through silver-associated oxidative and membrane damage.	158
AgNPs MGL-D10	Size: 30 ∼ 50 nm	AgNPs	—	*in vitro*	Supported antimicrobial coating development through Ag^+^-mediated membrane, enzymatic, and oxidative damage.	159
AgNPs	Size: 8.57 ± 1.17 nm (AgNPs), 4.01 ± 0.80 nm (AgNPs + AMP), 6.03 ± 0.87 nm (AgNPs + AMK); ζ: −40.80 ± 9.54 mV (AgNPs), −51.00 ± 20.20 mV (AgNPs + AMP), −21.10 ± 4.63 mV (AgNPs + AMK)	AgNPs; AMP; AMK	HFF	*in vitro*	Enhanced AMP/AMK activity against MDR uropathogens with low-dose cytocompatibility.	141
NDs	Size: 25 nm; ζ: −60 mV	NDs	T24	*in vitro*	Enhanced antibiotic-free intracellular UPEC clearance by targeting bladder epithelial reservoirs.	160
ZnO NPs	Size: 30 ∼ 50 nm	ZnO	—	*in vitro*	Demonstrated broad membrane/ROS-mediated activity against clinical UTI isolates.	161
SiO_2_-tannase nanoconjugate	Size: 15 nm	—	—	*in vitro*	Enhanced enzyme stability and bacterial wall penetration, improving inhibition of UTI pathogens.	162
CuS NPs	Size: 9 ∼ 20 nm	CuS	—	*in vitro*	Showed concentration-dependent antibacterial activity against multiple UTI pathogens via contact-mediated disruption.	104
CuO NPs	Size: 48 ± 4 nm	CuO/Cu^2+^	—	*in vitro*	Demonstrated antibacterial activity against clinical urinary pathogens through copper-mediated membrane and enzymatic damage.	163
Nla/Cn-CNPs	Size: 10 ∼ 60 nm	Nalidixic acid; curcumin	Vero	*in vitro*	Combined antibacterial, antibiofilm, antioxidant, and anti-inflammatory effects through multimechanistic action.	120

**Abbreviations:** AMK, amikacin; AMP, ampicillin; EAC, Ehrlich ascites carcinoma; ESBL, extended-spectrum β-lactamase; HFF, human foreskin fibroblasts; RBCs, red blood cells.

AgNPs are among the most extensively studied inorganic antibacterial materials [164]. They can exert bactericidal effects through Ag^+^ release, induction of ROS generation, disruption of bacterial membrane integrity, inhibition of DNA replication, and interference with key metabolic pathways, and therefore retain activity against many clinically relevant drug-resistant strains [165]. Srinivasan et al. [166] used an aqueous betel-leaf extract to synthesize AgNPs through a green approach and demonstrated that they inhibited quorum-sensing systems in the uropathogens *Serratia marcescens* and *Proteus mirabilis*, downregulated virulence- and biofilm-related gene expression, and exhibited favorable biocompatibility. Lopez-Carrizales et al. [141] combined AgNPs with AMP or amikacin (AMK) against multidrug-resistant uropathogens and showed synergistic antibacterial effects *in vitro*, with low cytotoxicity toward human fibroblasts at effective antibacterial concentrations (Fig. 5C). El-Batal et al. [158] prepared silver-boron binary inorganic nanoparticles (AgB NPs) by γ-irradiation-assisted green synthesis in the presence of polyvinylpyrrolidone (PVP) and found that they exhibited potent antibacterial and antibiofilm activity against multidrug-resistant uropathogens and fungi, suggesting that combined inorganic components may further strengthen bacteriostatic and membrane-disruptive effects.

Metal oxides and mesoporous inorganic materials have also been studied for UTI-related antimicrobial applications [167,168]. Zinc oxide nanoparticles may show favorable cytocompatibility under selected exposure conditions and warrant consideration for local anti-infective applications [169]. Hosseini et al. [157] treated clinical urinary isolates of *Candida albicans* with zinc oxide nanoparticles and found inhibition of early fungal adhesion, downregulation of ALS1 and ALS3, and activity against fluconazole-resistant strains. Mesoporous silica nanoparticles have large surface areas and ordered pores that support DL and controlled release [170]. Nsayef Muslim et al. [162] prepared silica nanoparticles by liquid-phase pulsed laser ablation and conjugated them with tannase from *S*. *marcescens*; *in vitro*, the enzyme-inorganic nanocomposite showed stronger inhibition of common uropathogens than conventional antibiotics such as CPF.

Compared with organic drug-delivery systems, inorganic nanomaterials in UTI emphasize the intrinsic antibacterial activity and physicochemical functions of the material itself. Mechanisms including metal-ion release, ROS generation, membrane disruption, enzyme-like catalysis, photothermal effects, and surface functionalization can suppress drug-resistant bacteria and biofilm formation without relying entirely on conventional antibiotic targets. However, these highly reactive properties also introduce translational risks, particularly local tissue toxicity, inflammation amplification, long-term accumulation, metabolic clearance, and perturbation of the urinary microbiome. Therefore, subsequent studies of inorganic nanoparticles should assess not only minimum inhibitory concentrations and antibiofilm efficacy, but also the therapeutic dose window, local urothelial biocompatibility, and long-term safety [98,171].

#### 4.1.3 Hybrid nanoparticles: Multifunctional integration and synergistic intervention

By integrating organic-carrier biocompatibility, inorganic-material functionality, and local-matrix retention, hybrid nanosystems offer broader design space for complex UTI settings. Table 3 summarizes representative hybrid platforms and highlights their multifunctional profiles. Compared with single-material systems, hybrid platforms can integrate antibacterial activity, antibiofilm effects, local retention or surface immobilization, controlled release, imaging, and anti-inflammatory regulation, making them suitable for device-associated infection, recurrent UTI with intracellular reservoirs, and combined anti-infective/anti-inflammatory therapy [172,173].

**Table 3:** Representative hybrid nanoparticle-based therapeutic platforms for UTI.

Nanoplatform	Particle characteristics	Active agent/Payload	Cell lines	Stage	Outcomes	Ref.
HBPAA-capped Ag@Au NPs	Size: 26.90 ∼ 41.68 nm; ζ: +31.64 mV	Ag@Au NPs	L929	*in vivo*	Achieved rapid contact killing and antibiofilm activity with reduced metal release.	142
SeNPs-CN	Size: 22.37 nm	GM	—	*in vitro*	Enhanced activity against MDR uropathogens and fungi, with improved biofilm inhibition.	174
PbAg NPs	Size: 20 ∼ 400 nm; PDI: 0.302; ζ: −23.1 mV	plant-derived capping components	PBMC, L132	*in vivo*	Suppressed quorum sensing, EPS production, motility, and biofilm formation.	166
HCu NPs	Size: 7 ∼ 12 nm	Cu^2+^	HeLa	*in vitro*	Exhibited broad-spectrum antibacterial activity against multiple UTI pathogens.	126
CMC-PVA-AgNPs	Size: 8 ∼ 14 nm	AgNPs	—	*in vitro*	Provided sustained AgNPs antibacterial activity in a green-synthesized hydrogel coating.	129
TET-BSA-NDs	Size: 43 nm (NDs), 76 nm (TET-BSA-NDs); PDI: 0.2; ζ: −70.5 mV (NDs), −33.4 mV (TET-BSA-NDs)	TET	T24	*in vivo*	Enhanced intracellular UPEC clearance via acidic endosomal TET release, while reducing systemic exposure.	105
D-xyl@εPLCDs	Size: 1.53 nm	εPL antimicrobial peptide-like polymer	5637, T24, J82, RAW264.7, HK-2, 2BS	*in vivo*	Reduced UPEC adhesion, invasion, intracellular survival, bladder burden, and inflammation.	175

**Abbreviations:** CMC, carboxymethyl cellulose; EPS, extracellular polymeric substances; HBPAA, hyperbranched polyamidoamine; PVA, polyvinyl alcohol; TET, tetracycline; εPL, ε-poly-L-lysine.

Gao et al. [142] embedded hyperbranched polyamidoamine-coated silver-gold core-shell nanoparticles in biodegradable PGA/PGLA ureteral stents, creating a contact-killing system with surface renewal. In a porcine urinary-tract model, this strategy reduced stent-associated infection and inflammation and offered a bioinspired approach for ureteral-stent-associated UTI (Fig. 5D). Emerging strategies, including antimicrobial-peptide-like nanomaterials and gene-editing nanodelivery systems, have also shown potential in basic UTI research [176,177]. Miao et al. [175] developed D-xylose-modified ε-poly-L-lysine carbon dots that inhibited UPEC adhesion, invasion, and intracellular survival at multiple stages through YadC-mediated targeting and antimicrobial-peptide-like membrane-disruptive effects. In an acute mouse UTI model, these carbon dots reduced bladder bacterial burden and inflammation. Gupta et al. [178] developed CRISPR-dot nanocomposites by using carbon quantum dots to deliver Cas9 and papG-targeting gRNA, thereby targeting the UPEC adhesion-associated virulence gene papG and reducing bacterial adhesion, biofilm formation, and pathogenicity.

Overall, hybrid nanoparticles and composite systems indicate a shift in UTI nanotherapy from single drug carriers toward multifunctional platforms. Their value lies in coordinated design that combines organic biocompatibility and controlled release, inorganic antibacterial or imaging functions, and surface modification or local immobilization. Future designs should be disease-setting specific: bladder infection requires urine stability and mucosal retention; intracellular infection requires cellular uptake and intracellular release; and device-associated infection requires durable coatings, anti-adhesive performance, and persistent antibiofilm activity. Hybrid systems also face manufacturing complexity, uncertain component interactions, additive toxicity, and batch inconsistency, requiring systematic standards for antibacterial mechanisms, safety assessment, and quality control [179].

### 4.2 Nanoparticles in CAUTI

CAUTI is among the clearest engineering applications of nanotechnology in inflammatory urinary disorders. Unlike non-catheter-associated UTI, CAUTI is strongly influenced by the catheter as an indwelling foreign body [16]. Urinary constituents, host proteins, and pathogens accumulate on the catheter surface, promoting bacterial adhesion, biofilm maturation, and crystalline encrustation. Thus, nano-enabled CAUTI prevention and control aim not only to increase antibacterial-drug concentrations in urine, but also to regulate the material–urine–microbe interface through catheter-surface functionalization (Fig. 6). Representative CAUTI nanoplatforms, including coating composition, payloads, model systems, and outcomes, are summarized in Table 4.

**Fig. 6:**
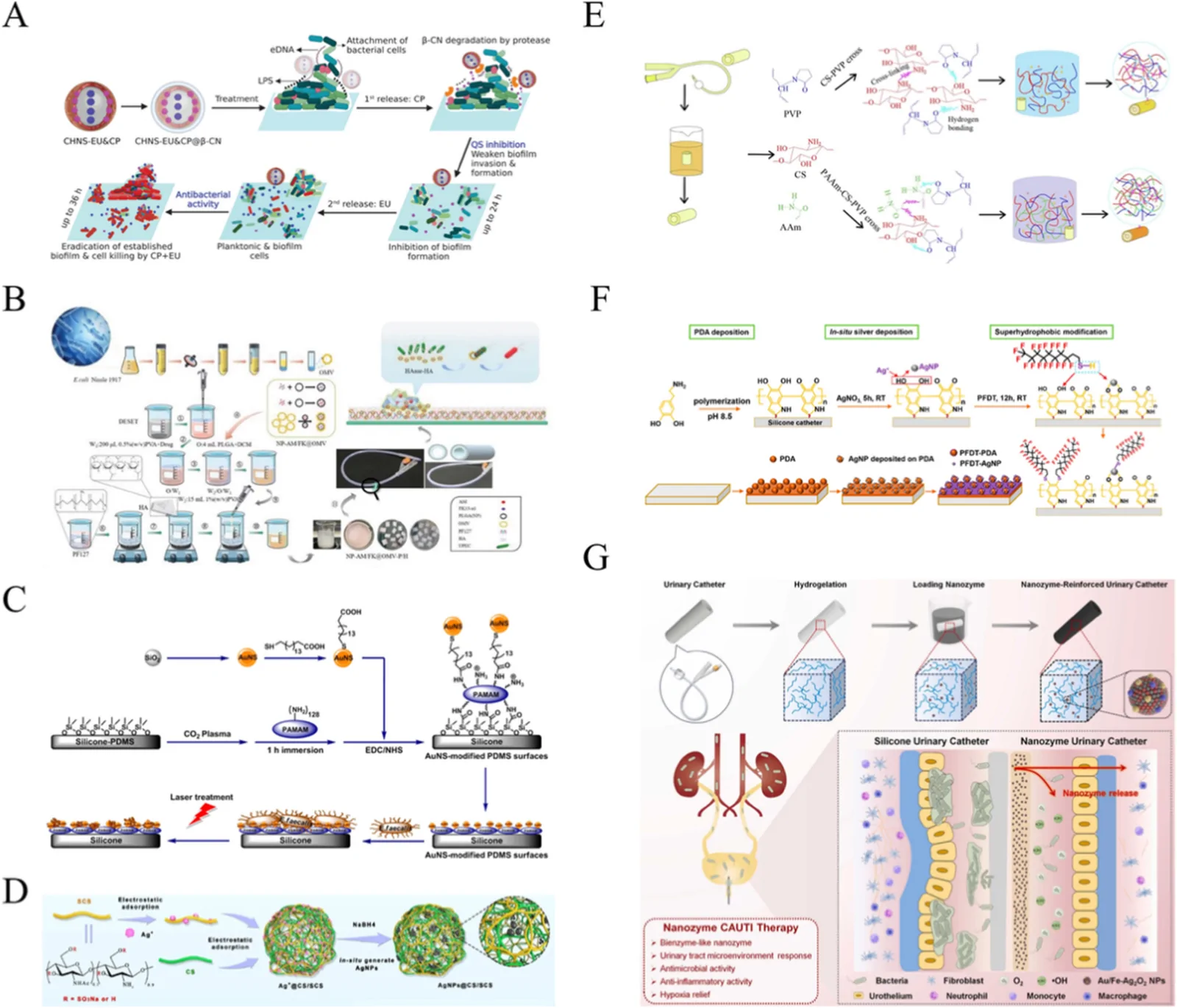
**Nanoparticle-based catheter interface engineering for CAUTI prevention and treatment. A:** CHNS capped with β-casein enable protease-triggered sequential release of a quorum-sensing inhibitor and bactericide for biofilm control. Reproduced with permission from Prateeksha et al. [180]. **B:** OMV-coated PLGA nanoparticles embedded in a Poloxamer/HA hydrogel co-deliver AMK and FK13-a1 for bacteria-responsive catheter protection. Reproduced with permission from Wu et al. [181]. **C:** Gold nanoshells immobilized on silicone surfaces generate local heat under NIR irradiation to eliminate adherent *Enterococcus faecalis*. Reproduced with permission from Khantamat et al. [182]. **D:** Sulfated chitosan/chitosan nanogels enable *in situ* AgNP formation and pH-dependent Ag^+^ release for sustained antibiofilm activity. Reproduced with permission from Fan et al. [183]. **E:** AgNPs incorporated into a PAAm-CS-PVP hydrogel improve catheter hydrophilicity and surface smoothness while providing sustained Ag^+^ release and antibacterial activity. Reproduced with permission from Cai et al. [184]. **F:** A PDA-AgNP-perfluorodecanethiol multilayer generates a superhydrophobic antibacterial surface that reduces adhesion, biofilm formation, and encrustation. Reproduced with permission from Zhang et al. [185]. **G:** Au/Fe co-doped silver peroxide nanozyme hydrogels integrate antibacterial, antibiofilm, oxygen-supplying, and anti-inflammatory functions. Reproduced with permission from Shang et al. [186]. **Abbreviations:** CHNS, chitosan hollow nanospheres; CS, chitosan; HA, hyaluronic acid; OMV, outer membrane vesicle; PAAm, polyacrylamide; PDA, polydopamine; PVP, polyvinylpyrrolidone.

**Table 4:** Representative organic and inorganic nanoparticle-based therapeutic platforms for CAUTI.

Material class	Nanoplatform	Particle characteristics	Active agent/Payload	Cell lines	Stage	Outcomes	Ref.
**Organic**	NP-AM/FK@OMV-P/H	Size: 100 ∼ 200 nm	AMK, DL: 9.20% ± 0.05%, EE: 82.94% ± 0.44%; FK13-a1 antimicrobial peptide, DL: 8.11% ± 0.34%, EE: 75.91% ± 3.21%;	3T3, HK-2, L02	*in vivo*	Enabled hyaluronidase-triggered release, improving anti-MDR and antibiofilm efficacy while limiting AMK toxicity.	181
	CHNS-EU&CP@β-CN	Size: 164.7 ± 0.72 nm; PDI: 0.21	Eugenol, EE: 44%; quorum-sensing inhibitor CP, EE: 38%	PCS-430-013	*in vivo*	Inhibited biofilm formation by >95% and prevented catheter occlusion for 30 days.	180
**Inorganic**	SME-AgNPs	Size: 29 nm; ζ: −43.6 mV	AgNPs	—	*in vitro*	Reduced *P. aeruginosa* virulence and biofilm formation at low concentrations.	187
	Zn_0_._12_Cu_0_._88_O	Size: 20 ∼ 95 nm	Zn-doped CuO NPs	L929	*in vivo*	Delayed CAUTI development by antibiotic-free biofilm inhibition in rabbits.	188
	AuNSs	Size: 185 ± 19 nm; ζ: −43.2 ± 0.6 mV	Au	—	*in vitro*	Eradicated adherent *E. faecalis* by NIR photothermal therapy with >1-month coating stability.	182
	FNPs	Size: 18 ∼ 20 nm; PDI: 0.236; ζ: −17.7 mV	L-fucose-functionalized AgNPs	293T, JHU011	*in vitro*	Improved *P. aeruginosa* antibiofilm activity at lower silver doses through LecB targeting.	189
	AgNPs	Size: 42 ∼ 75 nm	AgNPs; AMK; nitrofurantoin	—	*in vivo*	Suppressed UPEC catheter colonization *in vitro* and *in vivo*, with long-term coating stability.	127

**Abbreviations:** CHNS, chitosan hollow nanospheres; CP, quorum-sensing inhibitor; EU, eugenol; OMV, outer membrane vesicle.

#### 4.2.1 Organic nanoparticles and polymeric coatings: Interfacial antifouling, adhesion inhibition, and responsive controlled release

Organic nanoparticles and polymeric coatings in CAUTI prevention primarily regulate the catheter surface biointerface, reducing protein deposition, initial bacterial adhesion, and biofilm formation. After catheter insertion, urinary constituents and host proteins rapidly form a conditioning film that provides a substrate for bacterial adhesion and biofilm maturation [190]. Zwitterionic materials, chitosan nanostructures, and hydrogel coatings are therefore used to build antifouling, anti-adhesive, and controlled-release interfaces that extend catheter anti-infective function and reduce rapid depletion of conventional antibiotic coatings [191].

Composite designs based on zwitterionic polymers and antimicrobial peptides are representative of strategies that combine antifouling and contact killing. Ivanova et al. [192] used a one-step ultrasound-assisted green synthesis method to simultaneously prepare and deposit poly(sulfobetaine methacrylate)/polymyxin B hybrid nanoparticles on silicone urinary catheters. The zwitterionic polymer increased surface hydrophilicity and inhibited nonspecific protein adsorption, thereby reducing conditioning-biofilm formation and bacterial anchoring at the outset. Polymyxin B provided contact-killing activity against adherent or free *P. aeruginosa*. The coating significantly inhibited protein adsorption and *P. aeruginosa* biofilm formation, maintained stability in a simulated catheterization environment, and did not appreciably impair mammalian cell viability. Prateeksha et al. [180] constructed β-casein-capped hollow chitosan nanospheres co-loaded with a quorum-sensing inhibitor and a bactericidal agent. The system can be degraded by proteases in the bacterial-colonization microenvironment, triggering sequential release: the quorum-sensing inhibitor is released first to reduce bacterial surface hydrophobicity and the production of eDNA and LPS, thereby destabilizing biofilms; the bactericidal agent is subsequently released to enhance clearance of planktonic and biofilm-associated bacteria. Compared with antibacterial-agent-only or quorum-sensing-inhibition-only strategies, this system both suppressed biofilm formation and cleared established biofilms. In an artificial-urine flow model, coated catheters maintained antifouling capacity for an extended period, whereas uncoated catheters developed obvious biofilm obstruction within a short time (Fig. 6A).

Biologically derived nanocarriers combined with hydrogels provide another controlled-release strategy for CAUTI. Wu et al. [181] developed NP-AM/FK@OMV-P/H, a hyaluronidase-responsive nanoparticle–hydrogel coating for catheters. AMK and antimicrobial peptide FK13-a1 were loaded into PLGA nanoparticles, coated with probiotic-derived outer membrane vesicles (OMV), and embedded in a Poloxamer 407/HA hydrogel. The coating leveraged OMV interaction with UPEC, antibiotic–peptide synergy, and bacterial hyaluronidase-triggered release to limit premature drug depletion. Both *in vitro* and *in vivo*, it inhibited multidrug-resistant *E. coli* growth, biofilm formation, and catheter colonization, and reduced bacterial burden and inflammation in a mouse CAUTI model (Fig. 6B).

These studies indicate that organic nanoparticles and polymeric coatings are not merely drug reservoirs; rather, surface hydrophilization, antifouling modification, bacteria-responsive release, and multidrug delivery can delay bacterial adhesion and biofilm maturation on catheter surfaces. Compared with conventional antibiotic soaking or single-drug coatings, these systems may be more suitable for sustained prevention in long-term indwelling catheters. However, coating stability, release duration, and long-term biocompatibility under urinary flow, pH fluctuations, salt deposition, and mechanical friction require further validation [98].

#### 4.2.2 Inorganic nanoparticles: Metal-based antibacterial and antibiofilm activities

Unlike organic nanoparticles and polymeric coatings, which primarily rely on interfacial antifouling, adhesion inhibition, and controlled drug release, inorganic nanoparticles offer advantages in CAUTI prevention that derive largely from their intrinsic physicochemical activity. Metallic and metal oxide nanomaterials, such as silver, gold, zinc oxide, and copper oxide, can achieve antibacterial effects through metal-ion release, ROS generation, membrane damage, enzyme-like catalysis, or photothermal conversion [99]. Because catheter-surface biofilms are characterized by marked drug tolerance and persistent colonization, these materials are particularly suitable for engineering long-lasting antibacterial, antibiofilm, or externally triggered functional catheter surfaces [127,159].

AgNPs are widely investigated for antibacterial catheter modification, but their use is often constrained by the narrow balance between antibacterial efficacy and host-cell toxicity [193]. To address this issue, Bhargava et al. [189] functionalized AgNPs with L-fucose, enabling enhanced binding to *P. aeruginosa* PAO1 through L-fucose–LecB interactions and thereby improving bactericidal and antibiofilm effects at lower silver doses. The fucose-functionalized AgNPs exhibited greater antibacterial and antibiofilm activity than conventional citrate-coated AgNPs, an effect associated with increased ROS generation, oxidative membrane injury, and downregulation of virulence- and biofilm-related genes. They also markedly reduced bacterial colonization on artificial silicone surfaces, providing support for targeted functionalization of silver nanomaterials in CAUTI prevention. Inorganic photothermal nanostructures provide an alternative strategy for *in situ* eradication of catheter-adherent pathogens. Khantamat et al. [182] covalently modified PDMS model silicone catheters with carboxylated gold nanoshells, which generated local heat under 810-nm NIR irradiation. The coating markedly reduced adherent drug-resistant *E*. *faecalis*, and 10 min of irradiation substantially reduced colony formation with morphological evidence of thermal damage. (Fig. 6C).

Metal oxide nanomaterials can further provide synergistic prevention by combining antibacterial activity with disruption of the biofilm matrix [194,195]. Ivanova et al. [196] used a one-step sonochemical method to co-deposit zinc oxide nanoparticles and α-amylase on silicone Foley catheters, producing a ZnO@AM hybrid nanocoating. In this system, zinc oxide provided direct antibacterial activity, whereas amylase degraded extracellular polysaccharides in the biofilm matrix, thereby simultaneously targeting bacterial survival and biofilm structural stability. In a catheter model with artificial-urine circulation, the coating reduced biofilm formation by *S*. *aureus* and *E. coli* by approximately 60% and 80%, respectively, after 7 days under artificial urine flow. It also reduced bacteriuria and catheter-surface bacterial colonization in a rabbit CAUTI model without apparent cytotoxicity. Copper-based metal oxides likewise have strong antibacterial potential. Shalom et al. [188] prepared zinc-doped copper oxide nanoparticles and deposited them on urinary catheters to form a Zn-doped CuO nanocoating. The coating showed strong antibiofilm activity *in vitro* and delayed the onset of CAUTI in a rabbit catheterization model: infection appeared by day 4 in the uncoated-catheter group, whereas some animals receiving zinc-doped copper oxide-coated catheters did not develop infection until day 7 and others remained infection-free throughout the 7-day experiment. *In vivo* biocompatibility assessment did not reveal obvious local irritation or systemic toxicity.

These studies indicate that the value of inorganic nanocoatings in CAUTI lies primarily in intrinsic antibacterial activity, antibiofilm effects, and externally responsive killing. Compared with organic controlled-release coatings, they offer diverse mechanisms and persistent antibacterial effects that do not rely entirely on traditional antibiotics. However, clinical translation will require careful control of metal-ion release, long-term tissue compatibility, stability in the urinary environment, and safety boundaries under repeated triggering conditions.

#### 4.2.3 Hybrid nanoparticles and composite coatings: Multifunctional integration, long-term prevention, and device translation

Building on organic polymeric coatings and inorganic antibacterial nanomaterials, hybrid nanosystems integrate distinct functional modules. For long-term indwelling catheters, single antifouling, bactericidal, or controlled-release strategies often cannot simultaneously address protein deposition, repeated bacterial exposure, biofilm maturation, urine washout, and local inflammation [142]. Integrating metal nanoparticles, polymers, hydrogels, biomimetic adhesive layers, superhydrophobic structures, or nanozyme functions onto catheter surfaces has therefore become an important direction in nano-enabled CAUTI prevention. Table 5 summarizes hybrid coatings with antibacterial, antibiofilm, anti-encrustation, and biocompatibility outcomes.

**Table 5:** Representative hybrid nanoparticle-based therapeutic platforms for CAUTI.

Nanoplatform	Particle characteristics	Active agent/Payload	Cell lines	Stage	Outcomes	Ref.
Au/Fe-Ag_2_O_2_ NPs	Size: 70 nm; ζ: −6.8 ± 1.3 mV	Au/Fe co-doped Ag_2_O_2_ nanozymes	—	*in vivo*	Reduced catheter colonization, biofilm burden, and local inflammation *in vivo*.	186
AgNPs-PAAm-CS-PVP	Size: 22.98 ± 4.46 nm; PDI: 0.613	AgNPs	L929	*in vitro*	Maintained 30-day antibacterial activity with sustained Ag^+^ release and cytocompatibility.	184
ACPs@AgNPs	Size: 20 nm; ζ: −18.9 mV	AgNPs	HUVECs	*in vivo*	Provided 30-day antibacterial/antibiofilm activity with improved mechanical performance and biocompatibility.	197
ZnO@AM	Size: 192 nm; ζ: −22.7 ± 0.5 mV	ZnO NPs; α-amylase	HaCaT, BJ-5ta	*in vivo*	Maintained 7-day antibacterial and antibiofilm activity with favorable tissue compatibility.	196
PDA-AgNPs	Size: 100 ∼ 150 nm	AgNPs	L929	*in vitro*	Delayed catheter biofilm formation and encrustation via anti-adhesive surfaces and sustained Ag^+^ release.	185
Ag-PTFE	Size: ∼150 nm	Ag^+^	L929	*in vitro*	Combined Ag^+^-mediated antibacterial activity with low-energy anti-adhesive surface properties.	198
PDA-AgNPs	Size: 30 ∼ 50 nm	AgNPs	—	*in vitro*	Inhibited bacterial adhesion and biofilm formation through controlled 7-day Ag^+^ release.	199
ZnO@ZIF-8	Size: 80 ∼ 90 nm	ZnO within ZIF-8 framework	—	*in vitro*	Enabled scalable mature-biofilm removal through Zn^2+^/ROS-mediated contact killing.	200
AgNPs@CS/SCS	Size: 90 ∼ 225 nm; ζ: −25 mV	AgNPs	L929, 4T1	*in vivo*	Enabled pH-responsive 7-day Ag^+^ release with improved antibacterial, antibiofilm, and cytocompatibility outcomes.	183
PDA-AgNP	—	AgNPs	3T3	*in vitro*	Enabled tunable Ag^+^ release and reduced protein adsorption, bacterial adhesion, and biofilm formation.	201
TEL-AgNPs	Size: 20 ∼ 50 nm	Norfloxacin; TEL-Ag NPs/Ag^+^	—	*in vitro*	Provided early burst and ∼50-day antibacterial release while reducing urease-driven encrustation.	202

**Abbreviations:** ACPs, amphiphilic carbonaceous polymers; AM, α-amylase; CS, chitosan; PAAm, polyacrylamide; PDA, polydopamine; PTFE, polytetrafluoroethylene; PVP, polyvinylpyrrolidone; SCS, sulfated chitosan; TEL, triclosan-loaded elastic liposomes; ZIF-8, zeolitic imidazolate framework-8.

Organic-inorganic composites centered on AgNPs are among the most common designs in this area. Fan et al. [183] developed a chitosan/sulfated-chitosan nanogel system, AgNPs@CS/SCS, in which AgNPs were generated *in situ* within the gel network and Ag^+^ was released in a pH-dependent, sustained manner. Sulfated chitosan enhanced Ag^+^ binding and physiological stability, whereas the acidic environment associated with infection promoted Ag^+^ release, thereby combining short-term bactericidal and long-term antibiofilm effects (Fig. 6D). Cai et al. [184] further embedded AgNPs in a PAAm-CS-PVP composite hydrogel and coated both the luminal and external surfaces of latex urinary catheters by dip coating. In this system, AgNPs and chitosan provided synergistic antibacterial activity, while the PAAm-CS-PVP hydrogel improved surface smoothness, hydrophilicity, and lubricity, thereby reducing bacterial adhesion and prolonging antibacterial activity (Fig. 6E).

Beyond controlled-release composite hydrogels, combining structured surfaces with metallic antibacterial nanounits can suppress early colonization on catheter surfaces. Zhang et al. [185] constructed a PDA–AgNPs–perfluorinated thiol multilayer superhydrophobic coating on silicone urinary catheters by layer-by-layer self-assembly. The PDA layer anchored AgNPs *in situ*, followed by fluorinated low-surface-energy modification to create a micro/nanostructured superhydrophobic interface. Its anti-infective activity did not rely solely on Ag^+^ release; rather, it resulted from reduced bacterial contact and migration on the superhydrophobic surface, delayed biofilm formation by the micro/nanostructure, and sustained antibacterial activity provided by AgNPs. Compared with commercial silicone catheters and silver-alloy hydrogel catheters, the superhydrophobic coating markedly reduced adhesion of *E. coli* and *P*. *mirabilis*, delayed bacterial migration along the catheter, and extended the time to catheter obstruction from approximately 40 h to approximately 100 h, indicating strong potential for antibiofilm and anti-encrustation applications (Fig. 6F).

More recently, nanozyme-enhanced composite coatings have provided an additional multifunctional design for CAUTI prevention. Shang et al. [186] designed a hydrogel coating enhanced with gold/iron co-doped silver peroxide nanozymes for functional modification of silicone urinary catheters. The Au/Fe-Ag_2_O_2_ nanozyme served as the functional core, whereas the hydrogel acted as a hydrophilic loading matrix. Together, they endowed the catheter with both peroxidase-like and catalase-like activities: the former catalytically generated •OH to inhibit bacterial colonization and biofilm formation, whereas the latter generated oxygen, potentially alleviating local hypoxia and modulating immune-inflammatory responses. *In vitro* and *in vivo* results showed that the nanozyme-hydrogel coating achieved antibacterial, antibiofilm, and anti-inflammatory effects while improving catheter hydrophilicity and biocompatibility, representing an integrated strategy that moves CAUTI management from simple antibacterial activity toward coordinated antibacterial, anti-inflammatory, and microenvironmental modulation (Fig. 6G).

These studies indicate that hybrid nanocomposite coatings for CAUTI can integrate anti-adhesive effects, controlled antibacterial release, antibiofilm activity, anti-encrustation performance, and anti-inflammatory regulation on a single device surface. Compared with organic coatings, they provide more comprehensive functions; compared with single inorganic nanocoatings, their interfacial stability, biocompatibility, and durability may be more readily optimized through material combinations. Accordingly, CAUTI-directed nanotechnology has progressed from early antibacterial coatings that relied on a single bactericidal component toward multifunctional interface engineering for long-term indwelling catheters. Future work should further evaluate resistance to washout in urine, Ag^+^ release profiles and therapeutic windows for other active components, anti-encrustation capacity, long-term tissue compatibility, and scalable manufacturing and quality control to facilitate clinical translation of functionalized urinary catheters.

## 5 Nanoparticles in noninfectious inflammatory diseases of the urinary system

### 5.1 Nanoparticles in IC/BPS

IC/BPS has heterogeneous pathophysiology involving urothelial barrier injury, GAG-layer abnormalities, mast cell activation, local inflammation, oxidative stress, and neural sensitization [68]. Accordingly, nanoparticle applications in IC/BPS emphasize prolonged drug retention, mucosal interaction, barrier repair, and regulation of inflammation, oxidative stress, and pain-related pathways within the dynamic bladder environment (Fig. 7). Table 6 summarizes representative IC/BPS nanoplatforms across organic, inorganic, and hybrid material classes.

**Fig. 7:**
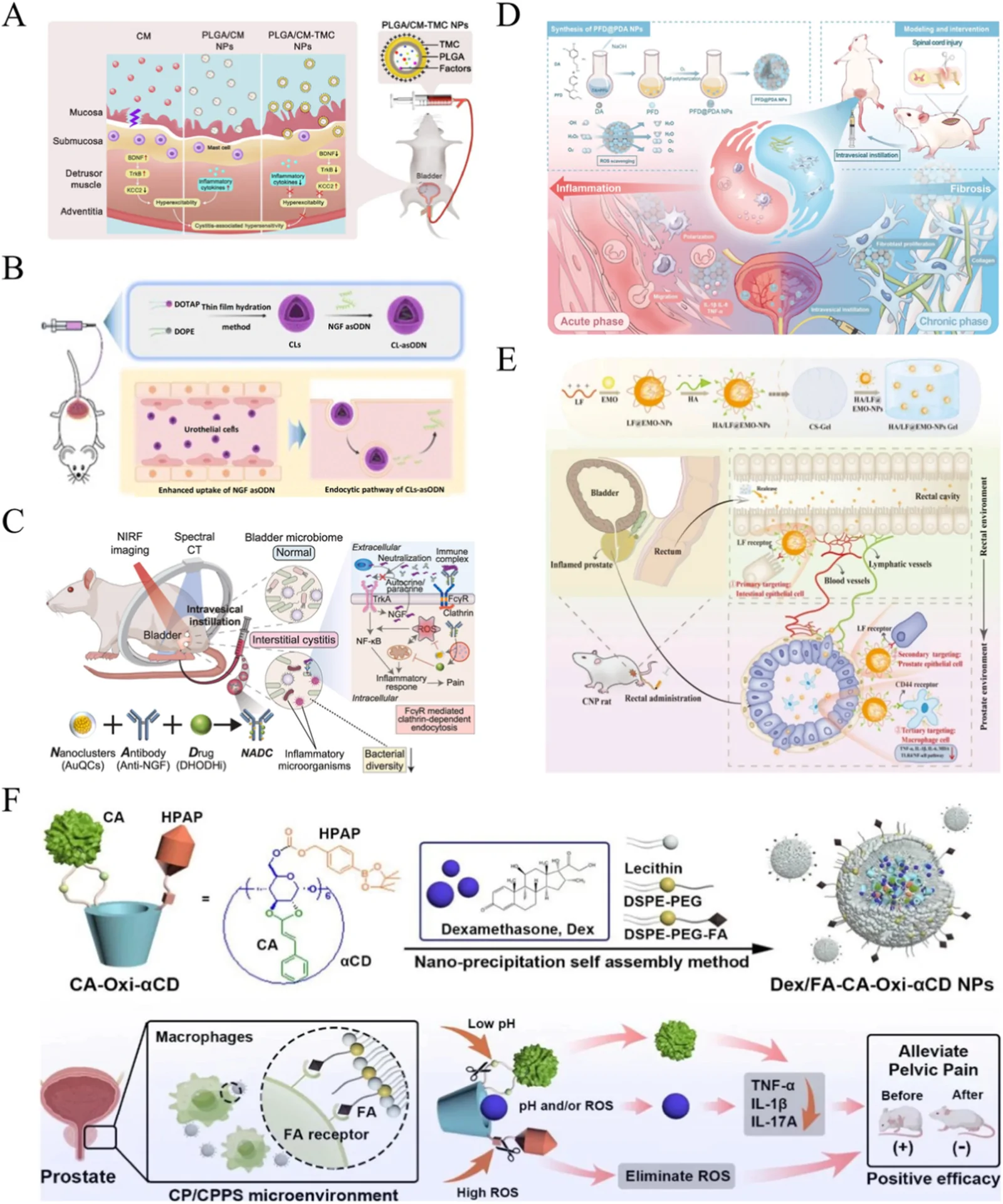
**Nanoparticle-based local delivery and mechanism-directed therapy for IC/BPS, CP/CPPS and related urological inflammatory disorders. A:** TMC-coated PLGA nanoparticles enhance intravesical delivery, urothelial adhesion, and transmucosal penetration of MSC-CM. Reproduced with permission from Lin et al. [203]. **B:** DOTAP/DOPE CLs deliver NGF-asODN to the bladder urothelium, suppressing NGF-related signaling and improving IC/BPS-associated dysfunction. Reproduced with permission from Gao et al. [204]. **C:** NGF-targeting NADC integrates anti-NGF antibody, AuQCs, and DHODHi for lesion-specific retention, NIRF/CT imaging, and IC/BPS therapy. Reproduced with permission from Lin et al. [205]. **D:** PFD@PDA nanoparticles adhere to the bladder wall, scavenge ROS, chelate Fe^2+^, protect mitochondria, suppress ferroptosis, and inhibit TGF-β-mediated fibrosis. Reproduced with permission from Zhang et al. [206]. **E:** HA/LF@EMO-NPs Gel combines LF-mediated epithelial targeting, HA–CD44-mediated macrophage targeting, and thermosensitive hydrogel retention for rectal delivery. Reproduced with permission from Ye et al. [207]. **F:** Dex/FA-CA-Oxi-αCD nanoparticles enable pH/ROS-responsive Dex release, ROS elimination, inflammatory-cell targeting, and pelvic-pain relief in CP/CPPS. Reproduced with permission from Yang et al. [208]. **Abbreviations:** asODN, antisense oligodeoxynucleotide; AuQCs, gold quantum clusters; CA, cinnamaldehyde; CLs, cationic liposomes; Dex, dexamethasone; DHODHi, dihydroorotate dehydrogenase inhibitor; EMO, emodin; LF, lactoferrin; MSC-CM, mesenchymal stem cell-conditioned medium; NADC, nanocluster-antibody-drug conjugate; NGF, nerve growth factor; NIRF, near-infrared fluorescence; PFD, pirfenidone; TGF-β, transforming growth factor-β; TMC, trimethyl chitosan.

**Table 6:** Representative nanoparticle-based therapeutic platforms for IC/BPS.

Material class	Nanoplatform	Particle characteristics	Active agent/Payload	Cell lines	Stage	Outcomes	Ref.
**Organic**	CLs/asODN	Size: 200 nm; ζ: +53 mV	NGF-asODN, EE >90%	SV-HUC-1	*in vivo*	Enhanced urothelial uptake and NGF silencing, improving urodynamic outcomes.	204
	LP-08	—	—	—	*in vivo*	Formed a protective urothelial film and alleviated pain, urgency, and frequency.	209
	Chitosan-TGA NPs	Size: 186 ± 6 nm; PDI: 0.29; ζ: +12 ± 1 mV	TMP, EE: 37%	—	*in vitro*	Prolonged bladder retention and sustained TMP release through thiol-mediated mucoadhesion.	114
	PLGA/CM-TMC NPs	Size: 375.3 nm; PDI: 0.19 ± 0.03; ζ: +19.8 ± 0.3 mV	MSC-CM, EE: 48.2 ± 3.1%	UC-MSCs, SV-HUC-1	*in vivo*	Enhanced urothelial adhesion and transmucosal delivery, improving pain, voiding, histology, and mast-cell infiltration.	203
	HA-PA/α-CD NPs	ζ: −37.3 ± 1.9 mV	α-cyclodextrin; HA	J774A.1, SV-HUC-1	*in vivo*	Reduced bladder inflammation and mucosal injury through GAG-replenishing and anti-inflammatory effects.	210
**Inorganic**	Cur-CONPs	Size: 150.88 ± 39.35 nm; ζ: −15.54 ± 4.96 mV	Curcumin, DL: 26.73%	T24	*in vivo*	Reduced inflammatory signaling, pelvic pain, voiding dysfunction, and urothelial barrier injury.	211
**Hybrid**	PFD@PDA NPs	Size: 157.90 ± 9.23 nm; PDI: 0.29 ± 0.02; ζ: −12.93 ± 0.95 mV;	PFD, DL: 26.5%	RAW264.7, SV-HUC-1, L-929	*in vivo*	Improved local retention and attenuated bladder inflammation, fibrosis, and dysfunction through ROS scavenging, mitochondrial protection, and ferroptosis suppression.	206
	Ur-PDA NCs	ζ: −23.5 ± 0.70 mV	—	RT4	*in vivo*	Achieved ∼60 μm bladder mucosal penetration through urea-powered self-propulsion and adhesion.	212
	NADC	Size: 28.2 ± 4.5 nm; ζ: −16.7 ± 0.7 mV	Anti-NGF antibody (tanezumab); DHODHi (vidofludimus)	SV-HUC-1, RAW264.7	*in vivo*	Enabled image-guided lesion targeting with improved inflammation, pain, voiding, and intravesical retention.	205

**Abbreviations:** asODN, antisense oligodeoxynucleotide; CLs, cationic liposomes; DHODHi, dihydroorotate dehydrogenase inhibitor; GAG, glycosaminoglycan; HA, hyaluronic acid; MSC-CM, mesenchymal stem cell-conditioned medium; NADC, nanocluster-antibody-drug conjugate; NGF, nerve growth factor; PA, palmitic acid; PFD, pirfenidone; TGA, thioglycolic acid; TMC, trimethyl chitosan; UC-MSCs, umbilical cord mesenchymal stem cells; α-CD, α-cyclodextrin.

#### 5.1.1 Organic nanoparticles: Intravesical retention and GAG-layer repair

Cyclic bladder filling and emptying, together with continuous urine dilution, cause conventional intravesical formulations to be rapidly eliminated after voiding, making it difficult to maintain stable local therapeutic concentrations [76]. Therefore, prolonging drug retention in the bladder lumen and at the urothelial surface is a central design objective for local delivery systems in IC/BPS. Organic nanoparticles with mucoadhesive properties show clear advantages in this regard. Materials such as chitosan nanoparticles, trimethyl chitosan (TMC)-modified polymeric nanoparticles, liposomes, and GAG-based self-assembled nanostructures exhibit favorable biocompatibility and can improve local delivery through electrostatic interactions, covalent bonding, mucosal penetration, or biomimetic barrier repair [213].

Mucoadhesion is a fundamental advantage of organic nanoparticles for intravesical drug delivery. Barthelmes et al. [114] prepared thiolated chitosan-thioglycolic acid nanoparticles by ionotropic gelation and loaded them with TMP as a model drug. The system exploited thiol groups to form disulfide bonds with cysteine-rich glycoproteins in the bladder mucosa, thereby enhancing covalent adhesion between nanoparticles and the bladder mucosa. In an *ex vivo* porcine bladder model under continuous artificial-urine washout, the thiolated chitosan nanoparticles exhibited approximately 14-fold greater adhesion than unmodified chitosan nanoparticles and showed a more sustained release profile in artificial urine. Polymeric nanoparticles have also been used to improve delivery of bioactive components into the bladder wall. Lin et al. [203] developed PLGA nanoparticles surface-modified with TMC for intravesical delivery of mesenchymal stem cell-conditioned medium (MSC-CM). PLGA provided a biodegradable carrier framework and protected protein and cytokine cargo, whereas TMC conferred a positive surface charge that enhanced interactions with the negatively charged urothelial surface. TMC modification enhanced urothelial adhesion, penetration, and local retention of the nanoparticles and, in a CYP-induced cystitis model, reduced mechanical hypersensitivity, improved voiding function, downregulated inflammatory cytokines, and modulated the BDNF/TrkB pain pathway (Fig. 7A). Liposomes are particularly suitable for delivering nucleic acid therapeutics and regulating specific molecular targets [214,215]. Nerve growth factor (NGF) plays an important role in pain sensitization and bladder dysfunction in IC/BPS, but NGF-antisense oligonucleotide (NGF-asODN) is susceptible to nuclease degradation and has limited transmembrane uptake. Gao et al. [204] developed cationic liposomes (CLs) to deliver NGF-asODN. Electrostatic interactions improved asODN encapsulation and stability, while CLs facilitated urothelial membrane interaction and uptake. The system achieved an asODN encapsulation efficiency of >90%, and urothelial uptake reached 14.6%, approximately 40-fold higher than that of free asODN. In a rat IC/BPS model, the formulation normalized voiding frequency and improved bladder capacity, compliance, and inflammation-related indices (Fig. 7B).

Beyond improving drug delivery, organic nanoparticles can be used to replenish and repair the damaged GAG layer. GAG-layer abnormalities are implicated in IC/BPS pathophysiology. GAG components such as HA, chondroitin sulfate, and heparin can prevent urinary irritants from entering the submucosa and help maintain urothelial barrier stability [216]. Although intravesical GAG replacement has been used clinically, free GAG solutions are readily diluted and eliminated by urine washout and voiding and have limited mucoadhesion and sustained activity [217]. Chuang et al. [209] conducted a prospective controlled study comparing intravesical liposome instillation with oral pentosan polysulfate sodium for IC/BPS. Intravesical liposomes showed favorable safety and improved urinary frequency, nocturia, pain, urgency, and O'Leary-Sant symptom scores, suggesting that liposomes can serve as local nanoplatforms for mucosal protection and barrier repair. Diaz-Salmeron et al. [210] further advanced GAG-replacement therapy by developing hydrophobically modified self-assembled nanosheets composed of HA, chondroitin sulfate, and heparin. The system formed flat hexagonal nanostructures through α-cyclodextrin-mediated inclusion of alkyl chains, thereby enhancing mucosal interaction and local retention of the GAG components. In a lipopolysaccharide (LPS)-induced macrophage inflammation model, HA nanosheets showed strong anti-inflammatory activity; in a rat IC/BPS model, they reduced bladder inflammation and promoted mucosal regeneration, with efficacy superior to that of native GAG solution and hydrophobically modified suspension.

The main value of organic nanoparticles in IC/BPS is to improve intravesical delivery and target barrier-injury-related pathology. With favorable biocompatibility, biodegradability, and surface modifiability, these platforms can enhance the adhesion, retention, and penetration of drugs at the urothelial surface and improve the local stability and utilization of proteins, nucleic acids, and GAG-based barrier-repair components. Organic nanosystems can also regulate key processes such as urothelial barrier disruption, inflammation, neural sensitization, and bladder hypersensitivity, thereby potentially prolonging local therapeutic effects, reducing repeat dosing, and limiting systemic adverse effects relative to conventional intravesical formulations.

#### 5.1.2 Inorganic nanoparticles: Oxidative-stress modulation and inflammatory microenvironment remodeling

Unlike the extensive use of inorganic nanomaterials for antibacterial activity, antibiofilm effects, and device-surface modification in UTI and CAUTI, the use of standalone inorganic nanoparticles in IC/BPS remains at an early stage. A representative direction is the use of inorganic nanomaterials with enzyme-like antioxidant activity to modulate the ROS-driven inflammatory microenvironment. Because oxidative stress contributes to urothelial barrier injury, inflammatory amplification, and pain sensitization, inorganic nanoplatforms capable of sustained ROS scavenging may provide a therapeutic approach to IC/BPS that is distinct from conventional anti-inflammatory drugs. Lin et al. [211] developed curcumin-loaded cerium oxide nanoparticles (Cur-CONPs) for IC/BPS therapy. Through the reversible Ce^3+^/Ce^4+^ redox cycle, cerium oxide provided sustained ROS scavenging, while curcumin enhanced short-term antioxidant and anti-inflammatory effects. *In vitro*, Cur-CONPs reduced intracellular ROS induced by 4-HC and downregulated IL-6, IL-1β, TNF-α, and COX-2. In a CYP-induced mouse model of IC/BPS, Cur-CONPs reduced voiding frequency and pelvic pain hypersensitivity and restored urothelial integrity.

Although current evidence remains limited, these findings suggest that the role of inorganic nanoparticles in IC/BPS should not be viewed solely through the antibacterial paradigm of metal-based nanomaterials used in infectious inflammatory diseases. Instead, greater emphasis should be placed on ROS regulation, barrier protection, and remodeling of the inflammatory microenvironment. Future studies should further clarify tissue retention, metabolic clearance, repeat-dosing safety, and long-term efficacy of inorganic nanomaterials such as cerium oxide after intravesical administration.

#### 5.1.3 Hybrid nanoparticles and local composite delivery systems: Multifunctional local therapy

Given the complexity of IC/BPS pathogenesis, single-function nanoplatforms are often insufficient to address its multifaceted pathological network. Accordingly, integrating mucoadhesion, anti-inflammatory activity, antioxidant effects, pain modulation, and GAG-layer repair within a single nanoplatform has become an important direction in IC/BPS nanotherapy.

Lin et al. [205] developed an NGF-targeting nanocluster-antibody-drug conjugate (NADC) that integrated an NGF-neutralizing antibody, a dihydroorotate dehydrogenase inhibitor (DHODHi), and ultrasmall gold quantum clusters (AuQCs) for intravesical theranostic intervention in IC/BPS. The antibody scaffold targeted NGF-high inflamed mucosa, AuQCs enabled NIR fluorescence and CT dual-modality imaging, and DHODHi provided immunomodulatory activity. After intravesical instillation, NADC showed prolonged retention in IC/BPS models and suppressed IL-17, NF-κB, TNF, and JAK-STAT pathways through NGF neutralization, FcγR-mediated macrophage uptake, and intracellular drug release. In chronic and acute IC/BPS models, as well as a prophylactic setting, NADC improved pain, urodynamic abnormalities, and inflammatory injury more effectively than dimethyl sulfoxide (DMSO) or HA (Fig. 7C). Zhang et al. [206] developed pirfenidone-loaded PDA nanoparticles (PFD@PDA NPs) for bladder inflammation and fibrosis after spinal cord injury. PDA enabled adhesion, mucosal penetration, ROS scavenging, iron chelation, and mitochondrial protection, whereas PFD inhibited TGF-β-related fibrosis. Although this study focused on neurogenic bladder rather than typical IC/BPS, its adhesive delivery and antioxidant-antifibrotic design provide useful principles for chronic inflammatory bladder disorders (Fig. 7D). Cell-derived extracellular vesicles (EVs) provide another biomimetic strategy for IC/BPS by combining natural intercellular delivery with immunomodulatory and regenerative activities. Song et al. [218] developed TGF-β3-induced MSC-derived EVs and showed that submucosal administration enhanced urothelial repair, suppressed inflammation, and restored bladder function in a chronic IC/BPS model, with superior therapeutic effects compared with MSC treatment.

In local composite delivery systems, hydrogels can serve as supporting matrices that prolong bladder residence and enable sustained release of therapeutic components [219]. Guo et al. [220] developed a composite decellularized-matrix/HA thermosensitive hydrogel (HA-Gel) using rabbit small-intestinal-submucosa-derived decellularized extracellular matrix as the thermosensitive scaffold and HA as a GAG-layer-repair component. The formulation remained liquid at room temperature, facilitating transurethral intravesical instillation, and was reported to undergo a sol–gel transition after administration, thereby forming a gel depot within the bladder and prolonging local retention. This study mainly established the material construction and formulation properties of HA-Gel, providing an important foundation for subsequent therapeutic evaluation in IC/BPS. Building on this platform, Liu et al. [221] further evaluated HA-Gel in a UPK3A65–84 peptide-induced rat model of IC/BPS. Using the same decellularized small-intestinal-submucosa matrix combined with HA for intravesical delivery, they found that 14 days of HA-Gel treatment significantly prolonged voiding intervals, increased maximum bladder capacity, and reduced mucosal edema, inflammatory-cell infiltration, mucosal shedding, and mast-cell infiltration. HA-Gel also downregulated inflammation- and pain-related molecules, including TNF-α, IL-6, and TRPM8, with most outcomes showing greater improvement than HA alone or gel alone. Notably, residues of the blank gel and HA-Gel remained detectable in the bladder for more than 14 days, suggesting strong retention and sustained activity. These findings suggest that HA-Gel may function not only as a sustained-release depot for HA, but also as a local therapeutic platform that improves IC/BPS pathology through GAG-layer repair, extracellular-matrix-scaffold-mediated mucosal remodeling, and suppression of immune-inflammatory responses.

Overall, hybrid nanoparticles and local composite delivery systems represent a shift in IC/BPS nanotherapy from single-drug loading toward intervention across multiple pathological processes. These platforms can integrate local retention, lesion targeting, therapeutic monitoring, and multimodal treatment functions to coordinately regulate barrier injury, inflammation, oxidative stress, neural sensitization, and fibrotic remodeling. Nanoparticle–hydrogel composite systems can form local drug depots in the bladder, more effectively resisting urine dilution and voiding-related washout while enabling sustained release. However, their long-term gel stability, degradation and clearance behavior, mucosal safety, and tissue responses after repeated instillation still require systematic validation. Future studies should further define the functional roles and safety boundaries of individual material components and, in conjunction with IC/BPS phenotyping, develop phenotype-informed local delivery platforms for patients with predominant barrier injury, inflammation, pain sensitization, or fibrotic remodeling.

### 5.2 Nanoparticles in CP/CPPS

The prostate is a glandular organ with distinctive anatomical and physiological features, including an acinar–ductal system, glandular epithelial tight junctions, local microcirculation, and the blood–prostate barrier, which collectively restrict effective drug diffusion into the prostatic interstitium, acini, and glandular lumina [222,223]. Following conventional systemic administration, drugs often fail to achieve stable, sustained, and sufficient therapeutic concentrations in inflamed prostatic tissue. Moreover, CP/CPPS itself is highly heterogeneous and involves chronic inflammation, oxidative stress, autoimmune dysregulation, neural sensitization, and pain amplification [83]. Thus, nanoparticles in CP/CPPS are valued not only for improving solubility or circulation time, but also for enhancing local prostatic accumulation, inflammatory-microenvironment-responsive release, immune modulation, attenuation of oxidative stress, and pain-related interventions (Fig. 7). Representative CP/CPPS platforms and related urological-pain nanotherapies are summarized in Table 7.

**Table 7:** Representative nanoplatforms for the treatment of urological and renal inflammatory diseases.

Disease	Nanoplatform	Physicochemical properties	Active agent/Payload	Cell lines	Stage	Outcomes	Ref.
**CP/CPPS**	HA/LF@EMO-NPs Gel	Size: 179.42 ± 1.56 nm; PDI: 0.209 ± 0.014	EMO, EE: 85.92 ± 1.34%	NCM-460, Caco-2, RWPE-1, RAW264.7	*in vivo*	Increased prostatic drug accumulation and reduced inflammation, fibrosis, oxidative stress, and pain.	207
	PLGA-PEMA-T2	Size: 400 ∼ 500 nm	T2 autoantigen peptide	—	*in vivo*	Induced antigen-specific immune tolerance, reducing pain, urinary frequency, and prostatic injury.	224
	Dex/FA-CA-Oxi-αCD NPs	Size: 179.63 ± 2.26 nm; PDI <0.2	Dex, DL: 6.5%	RAW264.7, MPECs, HPSCs	*in vivo*	Reduced inflammation, oxidative stress, pelvic pain, and depressive behaviors without evident systemic toxicity.	208
**UCPPS**	CNPs	Size: 93.2 nm; PDI: 0.25	—	T24	*in vivo*	Improved pain, voiding, edema, and urothelial integrity by ROS scavenging.	225
**Lupus nephritis**	DXM@G3DSP nanogel	Size: 100 ∼ 250 nm; ζ: +10 ∼ +70 mV	Dex; cfDNA-scavenging cationic nanogel	RAW264.7, BMDMs	*in vivo*	Reduced cfDNA/TLR9-driven inflammation, renal injury, immune-complex deposition, and mesangial proliferation.	226
**Glomerulonephritis**	A-DEX PEG-PLGA NPs	Size: 90 nm	Dex acetate	HBZY-1	*in vivo*	Enhanced renal cortical Dex exposure through mesangial-targeted delivery.	227
**Pyelonephritis**	Z-NPs	—	Zingerone	HEK293	*in vivo*	Lowered bladder and kidney bacterial burdens, oxidative stress, and inflammatory injury.	228
	QSINPs	Size: 685.7 nm; PDI: 0.5	Quorum-sensing inhibitor ajoene; chitosan/dextran sulfate polymeric carrier	—	*in vivo*	Inhibited *P. aeruginosa* quorum-sensing virulence and enhanced bacterial clearance with CPF.	229
**Ureteral stent-associated infection**	Heparin/PLL-Cu NPs	—	—	—	*in vivo*	Reduced bacterial adhesion, urease activity, urine alkalization, and stent encrustation.	230
**AKI**	rDONs@AuNR	Size: ∼90 × 60 nm (rDON), ∼48 × 9 nm (AuNR), ∼90 × 60 nm (dimer)	—	293T	*in vivo*	Reduced AKI biomarker levels and tubular injury through ROS scavenging.	231
	HA/SS-31/CS	Size: 53 ± 0.17 nm; PDI: 0.206 ± 0.06; ζ: −19.6 ± 0.7 mV	SS-31 mitochondria-targeted antioxidant peptide, DL: 10.5 ± 0.2%, EE: 94.0 ± 2.0%	HUVECs	*in vivo*	Reduced renal dysfunction, oxidative stress, inflammation, and apoptosis through lysosomal SS-31 release.	232

**Abbreviations:** AKI, acute kidney injury; AuNR, gold nanorod; BMDMs, bone marrow-derived macrophages; CA, cinnamaldehyde; cfDNA, cell-free DNA; Dex, dexamethasone; EMO, emodin; FA, folic acid; HPSCs, human prostate stromal cells; LF, lactoferrin; MPECs, mouse prostate epithelial cells; Oxi-αCD, oxidation-responsive α-cyclodextrin; PEG, polyethylene glycol; PEMA, poly(ethylene-alt-maleic anhydride); PLL, poly-L-lysine; QSINPs, quorum-sensing inhibitor nanoparticles; rDONs, rectangular DNA origami nanostructures; SS-31, Szeto–Schiller peptide 31; TLR9, Toll-like receptor 9; UCPPS, urological chronic pelvic pain syndrome.

Rectal administration is a potentially relevant local delivery route for CP/CPPS. Because the rectum is anatomically adjacent to the prostate, rectally absorbed drugs may access periprostatic tissues through local diffusion and perirectal venous and lymphatic networks, increasing local exposure while reducing systemic burden [233,234]. Ye et al. [207] developed emodin (EMO)-loaded lactoferrin/HA-functionalized nanoparticles with multitargeting capability and incorporated them into a chitosan/β-glycerophosphate thermosensitive hydrogel (HA/LF@EMO). LF targeted intestinal and prostatic epithelial cells, HA recognized CD44-expressing macrophages at inflamed sites, and hydrogel embedding enhanced rectal retention and sustained release (Fig. 7E).

For systemic delivery, inflammatory-microenvironment-responsive nanomedicines can enhance local drug accumulation and selective release in inflamed prostatic tissue. Yang et al. [208] developed a pH/ROS dual-responsive dexamethasone (Dex) nanoformulation using a cinnamaldehyde-modified α-cyclodextrin carrier and folate surface modification to enhance uptake by folate-receptor-expressing macrophages and prostatic cells. In acidic and ROS-rich inflammatory microenvironments, the formulation underwent pH/ROS-responsive Dex release, reduced ROS, and downregulated TNF-α, IL-1β, and IL-17A. *In vivo*, it accumulated in prostatic tissue, attenuated pelvic pain hypersensitivity, reduced prostatic inflammation, and was associated with reduced depression-like behavior in mice (Fig. 7F).

Nanoparticles can also induce immune tolerance to address potential autoimmune dysregulation in CP/CPPS. Cao et al. [224] developed an antigen-coupled immune-tolerance nanoplatform by conjugating the CP/CPPS-relevant T2 peptide, a dominant epitope of TRPM8, onto PLGA-PEMA nanoparticles. In a T2-peptide-induced mouse model, PLGA-PEMA-T2 increased pelvic pain thresholds, reduced voiding frequency and inflammatory-cell infiltration in the prostate, decreased TNF-α and C-reactive protein, and increased IL-10. These findings indicate that antigen-conjugated nanoparticles may ameliorate CP/CPPS phenotypes through immune-tolerance induction, providing a strategy distinct from conventional anti-inflammatory or analgesic therapy for CP/CPPS with autoimmune features.

Direct evidence for inorganic nanoparticles in CP/CPPS remains scarce, but their antioxidant and immunomodulatory effects in UCPPS models are informative. Lien et al. [225] pretreated a CYP-induced UCPPS mouse model with cerium oxide nanoparticles and found that they alleviated pelvic pain, urinary frequency, and bladder inflammatory injury by scavenging ROS and downregulating inflammation-associated signals such as SerpinB2 and CXCL10. Although this work focused on UCPPS rather than CP/CPPS, it suggests that inorganic antioxidant nanomaterials can ameliorate chronic-pelvic-pain-related pathology through modulation of the ROS-inflammation axis and provides a rationale for future development of nanozyme or ROS-scavenging platforms in the prostatic inflammatory microenvironment.

Nanotherapy for CP/CPPS remains preclinical, with evidence largely limited to cell and animal studies. Several directions have emerged: local composite systems may support sustained rectal administration; responsive organic nanoparticles can enhance local accumulation, controlled release, and the safety profile of anti-inflammatory therapy; antigen-conjugated nanoparticles may induce antigen-specific immune tolerance; and inorganic antioxidant nanomaterials may support ROS scavenging and inflammatory-microenvironment remodeling. Future studies should align material composition, administration route, targeting strategy, and long-term safety with phenotype-informed frameworks based on predominant inflammation, autoimmune features, neural sensitization, or fibrotic remodeling.

## 6 Nanoparticles in other urological and renal inflammatory disorders

Beyond UTI, CAUTI, IC/BPS, and CP/CPPS, nanoparticles have also attracted attention in other inflammatory urinary disorders and inflammation-related settings. These areas are supported by fewer studies and greater disease heterogeneity, including upper UTI, device-associated infections, renal immune inflammation, and acute kidney injury (AKI) [103,235]. The corresponding platforms, physicochemical properties, payloads, models, and outcomes are summarized in Table 7. These studies show that nanoplatforms are not confined to lower-urinary-tract delivery or antibacterial therapy, but can be extended to antibiofilm treatment, anti-encrustation, kidney-targeted delivery, immune regulation, ROS scavenging, and theranostics (Fig. 8).

**Fig. 8:**
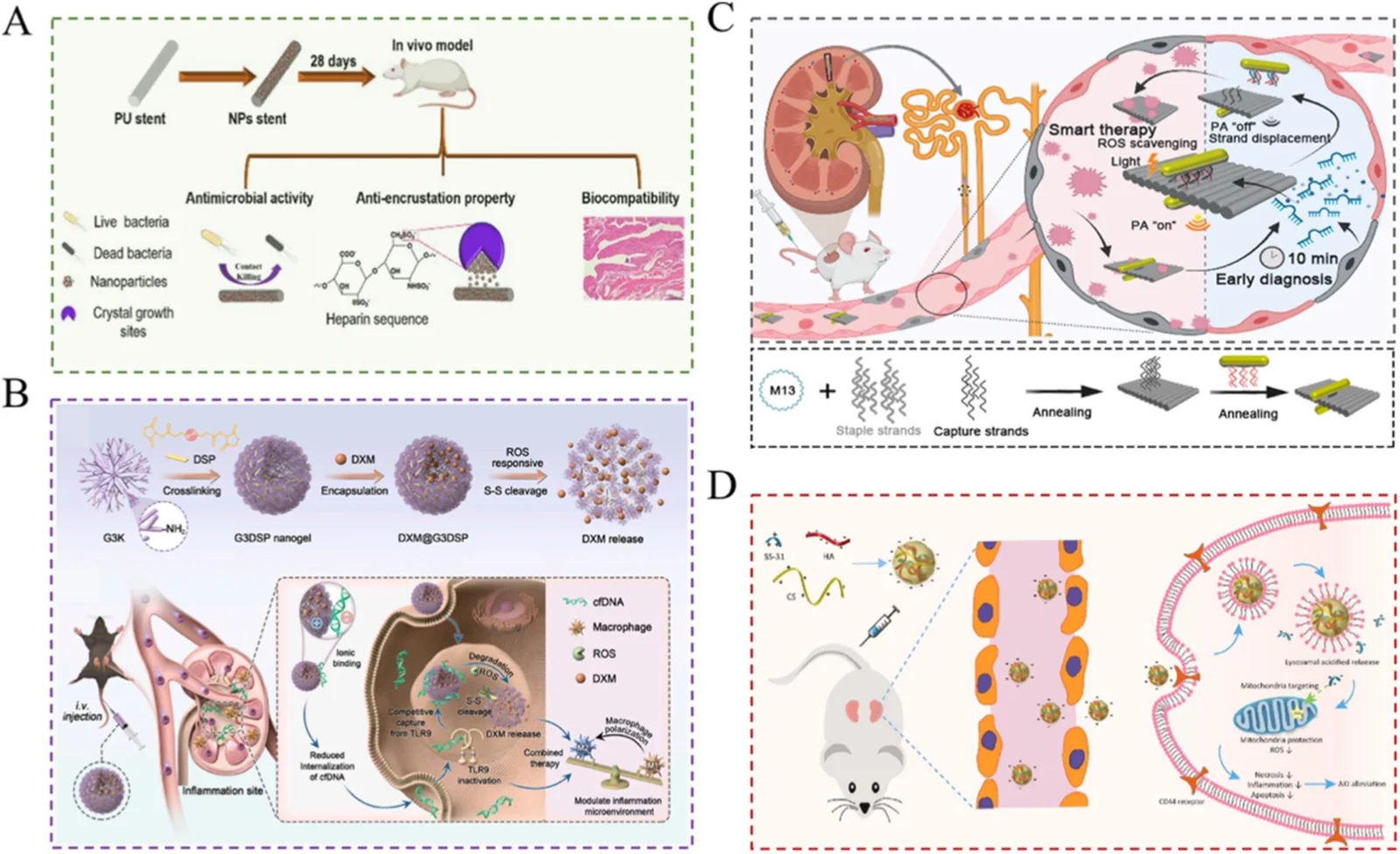
**Nanoparticle-enabled local interface engineering and kidney-targeted therapy for urinary tract and renal inflammatory disorders. A:** Heparin/poly-L-lysine/copper nanoparticle coatings endow ureteral stents with antibacterial and anti-encrustation functions. Reproduced with permission from Awonusi et al. [230]. **B:** DXM@G3DSP nanogels enable renal homing, cfDNA scavenging, and ROS-responsive Dex release for lupus nephritis. Reproduced with permission from Zhu et al. [226]. **C:** rDONs@AuNR nanoantennas provide AKI-responsive imaging and ROS-scavenging DNA-origami therapy. Reproduced with permission from Xu et al. [231]. **D:** HA/SS-31/CS nanopolyplexes enhance AKI-kidney accumulation via HA–CD44 targeting and release SS-31 for mitochondrial protection, ROS reduction and attenuation of tubular injury. Reproduced with permission from Liu et al. [232]. **Abbreviations:** AKI, acute kidney injury; AuNR, gold nanorod; cfDNA, cell-free DNA; rDONs, rectangular DNA origami nanostructures; SS-31, Szeto–Schiller peptide 31.

Acute pyelonephritis is an upper UTI involving the renal pelvis and parenchyma and is commonly accompanied by inflammatory kidney injury. Compared with lower UTI confined to the bladder, pyelonephritis nanotherapy emphasizes reducing renal bacterial burden, overcoming biofilm-associated antibiotic tolerance, and alleviating infection-induced inflammation [236,237]. Sharma et al. [228] developed zingerone-loaded chitosan nanoparticles (Z-NPs) for *P. aeruginosa* biofilm-associated acute pyelonephritis in mice. Z-NPs showed cytocompatibility in renal cells, reduced kidney and bladder bacterial burdens, decreased malondialdehyde (MDA), myeloperoxidase (MPO), and reactive nitrogen intermediates (RNI), improved histopathology, and enhanced serum bactericidal activity, phagocytic activity, and intracellular killing. Vadekeetil et al. [229] loaded a garlic-derived quorum-sensing inhibitor into chitosan/dextran sulfate nanoparticles (QSINPs) and administered them intravesically in a model of *P. aeruginosa* biofilm-associated pyelonephritis. The formulation reduced virulence-factor production, including elastase, protease, rhamnolipids, and alginate, inhibited biofilm formation, and, with CPF, further reduced bladder and kidney bacterial burdens.

Ureteral stent-associated infection, encrustation, and local inflammation represent another device-related urological inflammatory situation beyond CAUTI. Stents are exposed to urine for prolonged periods and are prone to bacterial adhesion, biofilm formation, and crystalline encrustation, leading to obstruction, impaired urine flow, pain, persistent infection, or sepsis. Awonusi et al. [230] immobilized heparin/poly-L-lysine-copper nanoparticles on polyurethane ureteral stents and evaluated them in a rat model of infectious cystitis. The nanocoated stents reduced bacterial adhesion, bacterial urease activity, urinary alkalinization, calcium and magnesium crystal deposition, and tissue inflammatory responses after 28 days, suggesting that copper-containing nanocomposite coatings can improve ureteral-stent-associated infection through sustained antibacterial activity and reduced crystal deposition (Fig. 8A).

In renal immune-inflammatory diseases, nanoparticle use differs from that in lower urinary tract disorders. The goal is not intraluminal retention, but targeted delivery to glomeruli, mesangial regions, renal tubules, or inflamed kidney tissue. Li et al. [227] exploited the size-selective permeability of glomerular endothelial fenestrae to design 90-nm PEG-PLGA nanoparticles loaded with Dex acetate for mesangial-region delivery. These nanoparticles accumulated more effectively in the kidneys, particularly the renal cortex, than free drug and colocalized with mesangial-cell markers while maintaining stability and sustained release, providing proof of concept for targeted glucocorticoid delivery in mesangial proliferative glomerulonephritis. Lupus nephritis is a renal immune-inflammatory disorder driven by immune complexes, extracellular nucleic acids, and renal inflammation. Zhu et al. [226] developed dexamethasone-loaded DNA-scavenging nanogels (DXM@G3DSP) for lupus nephritis associated with systemic lupus erythematosus. The cationic polylysine dendritic nanogel sequestered and cleared cell-free DNA (cfDNA), suppressing TLR9-mediated immune activation, preferentially accumulated in inflamed kidneys, and enabled ROS-responsive DXM release. *In vivo*, it reduced cfDNA accumulation, improved indices of renal function, and attenuated systemic inflammation, illustrating a multifunctional strategy integrating pathogenic-nucleic-acid scavenging, immunosuppressive drug delivery, and inflamed-kidney targeting (Fig. 8B).

Although AKI is not a classical urological inflammatory disorder, AKI induced by ischemia–reperfusion, sepsis, or nephrotoxic drugs is characterized by pronounced oxidative stress, mitochondrial damage, inflammatory-cell infiltration, and tubular epithelial-cell apoptosis and necrosis. Therefore, it also represents an important extension of nano-enabled anti-inflammatory and antioxidant therapy. Cell-membrane coating provides a complementary biomimetic approach for renal inflammatory injury. Liu et al. [238] developed neutrophil-membrane-enveloped CoQ10 nanoparticles (N-NP), in which the neutrophil membrane conferred inflammation-associated interactions while the nanoparticle core delivered the antioxidant payload. In a renal ischemia–reperfusion model, N-NP reduced oxidative stress, inflammatory cytokine release, apoptosis, and tubular injury and improved renal function. Xu et al. [231] designed a miR-21-responsive DNA-origami nanoantenna for early diagnosis and responsive therapy of AKI. The system assembled small gold nanorods on a DNA-origami scaffold to form a NIR-II photoacoustic nanoantenna that preferentially accumulated in kidneys. In miR-21-rich early AKI, the nanoantenna disassembled and altered the photoacoustic signal, enabling early imaging-based diagnosis; released DNA-origami scaffolds scavenged ROS and alleviated oxidative kidney injury (Fig. 8C).

Liu et al. [232] developed a HA/chitosan pH-responsive nanocomposite for the mitochondrial antioxidant peptide SS-31. The system used HA–CD44 interactions to enhance accumulation in injured kidneys and released SS-31 in acidic endolysosomal compartments. Compared with free SS-31, it more effectively reduced serum creatinine and blood urea nitrogen and attenuated tubular necrosis, mitochondrial damage, ROS accumulation, inflammatory cytokine expression, and apoptosis (Fig. 8D).

Collectively, these studies suggest that nanoparticle applications beyond the four major disorders are strongly context dependent. For upper UTI, including pyelonephritis, nanoplatforms mainly enhance antibacterial, antivirulence, antibiofilm, and host-defense effects. For ureteral stent-associated infection and encrustation, nanocoatings emphasize anti-adhesive activity, anti-encrustation performance, and local inflammation control at device surfaces. For renal inflammatory diseases such as glomerulonephritis, lupus nephritis, and AKI, nanotherapy relies more on kidney targeting, immune regulation, nucleic-acid scavenging, ROS-responsive release, and mitochondrial protection. Although most approaches remain at the animal-model or early materials-validation stage, they broaden the scope of potential nanoparticle applications and highlight the need to tailor material composition, particle size, surface modification, and administration route to disease location, inflammation type, and dominant pathological drivers.

## 7 Discussion

### 7.1 Current status and advantages of nanoparticles in the treatment of inflammatory diseases of the urinary system

This review summarizes nanoparticle-based therapeutic approaches for major inflammatory diseases of the urinary system, including UTI, CAUTI, IC/BPS, and CP/CPPS. The field has evolved from carrier strategies focused on antibiotic delivery toward integrated platforms combining antibacterial, antibiofilm, anti-inflammatory, antioxidant, mucosal-repair, immunomodulatory, pain-modulating, and stimuli-responsive drug-release functions. Beyond improving drug solubility, half-life, and encapsulation efficiency, nanosystems can modulate local drug accumulation, tissue penetration, mucosal retention, release kinetics, and multitarget mechanisms of action, thereby better accommodating the dynamic pathological microenvironment of inflammatory urinary disorders.

Infectious inflammatory diseases currently represent a relatively mature area of nano-enabled therapeutic research. The principal barriers in UTI and CAUTI treatment include bacterial adhesion, biofilm formation, and antibiotic resistance; intracellular bacterial reservoirs are particularly relevant to recurrent UTI. Nanoparticles can address these barriers by increasing local antibiotic concentrations, enhancing biofilm penetration, promoting intracellular delivery, providing metal-ion- or ROS-mediated multimodal killing, and constructing anti-adhesive controlled-release coatings [15,128]. CAUTI provides a well-defined engineering setting through urinary-catheter surface modification. Silver-, copper-, and zinc oxide-based materials, nanozymes, polymeric nanocoatings, and nanoparticle–hydrogel composite coatings have shown antibacterial, antibiofilm, and anti-encrustation potential. Some silver-alloy or antimicrobial-coated catheters have entered clinical use or evaluation, indicating an initial translational basis for nanomaterials in urinary infection prevention [56]. Nanotherapy for noninfectious chronic inflammatory disorders such as IC/BPS and CP/CPPS remains earlier in development, but its mechanism-guided value is important. IC/BPS approaches emphasize bladder mucosal retention, GAG-layer repair, inflammation and oxidative-stress regulation, and pain-related pathways such as NGF. CP/CPPS strategies emphasize prostatic-barrier penetration, rectal delivery, inflammatory-microenvironment-responsive release, immune modulation, and attenuation of pelvic pain. Hydrogels, exosomes/EVs, and immune-cell-related nanoplatforms have also been introduced [76,239,240]. Hydrogels can serve as local depots for intravesical instillation, catheter coatings, or rectal delivery; EVs can deliver miRNAs, proteins, or anti-inflammatory signals [218]; and immune-cell-membrane-coated nanoparticles can exploit inflammation-homing and immunomodulatory properties to improve lesion accumulation and biocompatibility [241,242].

The advantages of nanoparticles can therefore be summarized at three levels: (1) delivery, by improving drug stability, local exposure, tissue penetration, and controlled release; (2) materials, by exploiting intrinsic antibacterial, antioxidant, adhesive, or barrier-repair properties; (3) mechanisms, by integrating anti-infective, anti-inflammatory, immunomodulatory, oxidative-stress-control, antifibrotic, and pain-pathway interventions. Nanotherapy should not be viewed simply as an upgraded formulation, but as a platform for reshaping local treatment and pathological-microenvironment intervention.

### 7.2 Limitations of nanoparticles in the treatment of inflammatory diseases of the urinary system

Although nanoparticles show broad potential in inflammatory urinary disorders, current evidence derives mainly from *in vitro* experiments and short-term animal studies. Clinical translation also depends on disease-model validity, long-term safety, manufacturing consistency, feasible administration, and clinically relevant endpoints (Fig. 9).

**Fig. 9:**
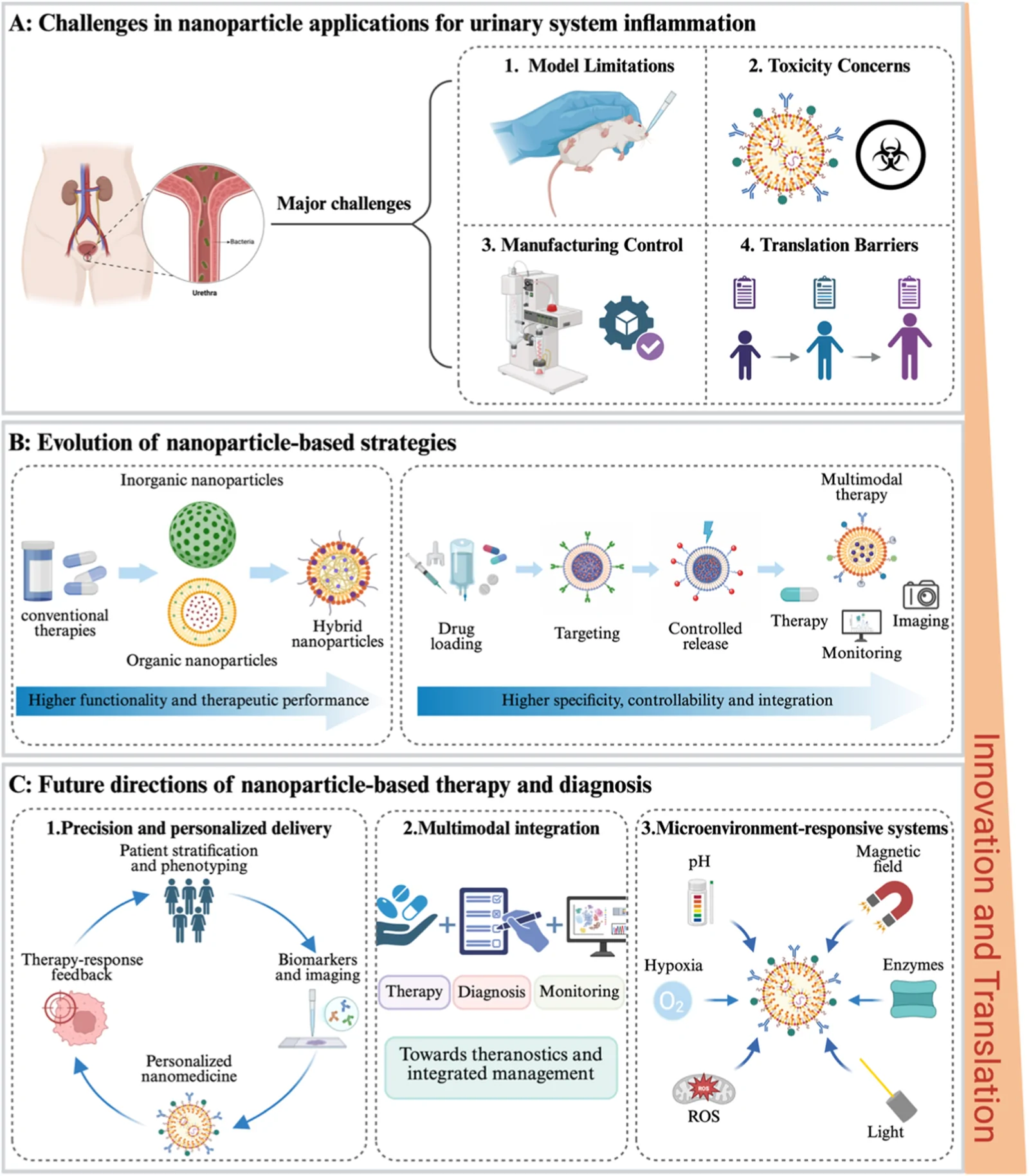
**Challenges, strategic evolution, and future directions of nanoparticle-based therapeutic platforms for urinary system inflammation. A:** Major challenges include inadequate disease-model fidelity, potential systemic toxicity, insufficient manufacturing and quality control, and translational barriers. **B:** Nanoparticle strategies are evolving from conventional drug loading toward active targeting, controlled release, and integrated therapy–imaging–monitoring functions. **C:** Future development should prioritize personalized delivery, multimodal theranostic integration, and microenvironment-responsive systems. Created with BioRender.com.

#### 7.2.1 Limited fidelity of preclinical disease models

Current models of inflammatory urinary disorders include transurethral bacterial inoculation, catheter implantation, CYP induction, autoimmune induction, and local tissue injury. These models can reproduce selected pathological elements of infection, acute inflammation, or mucosal injury, but generally cannot fully capture the chronicity, recurrence, and heterogeneity of clinical disease [239,240,243].

For UTI, commonly used models rely on single transurethral UPEC inoculation followed by short-term assessment of urine or bladder bacterial burden, inflammatory mediators, and histopathology. These models are suitable for acute infection control but do not determine whether IBCs or quiescent intracellular reservoirs have been eliminated. Short-term negative urine cultures or reduced bladder colony counts are therefore insufficient to prove durable recurrence prevention. Long-term follow-up, rechallenge experiments, intracellular bacterial quantification, and recurrence-rate evaluation remain underused [244]. CAUTI models have similar limitations: many use static cultures, silicone coupons, or short-term catheter implantation and omit continuous urinary flow, conditioning biofilms, protein deposition, pH fluctuations, urease-positive encrustation, and polymicrobial biofilms. Single *E. coli* models may not reproduce the obstruction and complex biofilms associated with *Proteus* spp., *P. aeruginosa*, or *Candida* spp. Long-term coating integrity, material shedding, catheter patency, and urothelial compatibility also require evaluation [244].

Model extrapolation is even more difficult for noninfectious chronic disorders such as IC/BPS and CP/CPPS [245]. CYP-induced cystitis reproduces acute urothelial injury, inflammation, and bladder hypersensitivity but not long-term neural sensitization, immune imbalance, or clinical phenotypic diversity [246]. In prostatitis research, bacterial-inoculation models mainly reflect infection-related pathology, whereas experimental autoimmune prostatitis models primarily reflect immune dysregulation. Neither captures pelvic-floor dysfunction, gut microbiota alterations, peripheral sensitization, or central pain sensitization in CP/CPPS [247]. Thus, short-term anti-inflammatory or analgesic effects in a single model should be extrapolated cautiously to chronic clinical disease.

#### 7.2.2 Insufficient long-term safety evaluation

Long-term safety is a key issue for the clinical translation of nanodelivery systems for inflammatory urinary disorders. The sources of risk differ substantially across material platforms, and short-term cell-viability assays or single histological observations are generally insufficient to reflect local tolerability and systemic exposure risks under repeated dosing, long-term indwelling use, or urothelial mucosal injury [[248], [249], [250], [251]].

For inorganic nanomaterials such as silver, copper, gold, zinc oxide, copper oxide, and cerium oxide, antibacterial, antibiofilm, antioxidant, or imaging effects may depend on metal-ion release, ROS modulation, surface catalysis, or optical properties [252]. These effects are dose dependent. Excessive metal-ion release may impair urothelial viability, redox homeostasis, and mucosal-barrier integrity; in cystitis, catheter irritation, or epithelial injury, systemic distribution and organ accumulation of particles or degradation products may increase [253]. For ROS-generating antibacterial materials, the therapeutic window between bacterial killing and urothelial oxidative injury should be defined, and long-term effects on major organs should be assessed [251,254].

Liposomes, PLGA, chitosan, and other polymeric nanocarriers generally exhibit favorable biocompatibility, but their degradation products, particle-size distribution, surface charge, and repeat-dosing regimen may still influence local tissue tolerance. In particular, highly cationic materials can enhance mucoadhesion and cellular uptake but may also perturb cell membranes or mucus-layer structure and provoke local inflammatory responses [255]. For intravesical systems, evaluation should extend beyond histological changes after a single administration to include urothelial integrity, barrier function, bladder-irritation symptoms, carrier residues, and drug clearance after repeated dosing. For rectal or urethral local delivery systems, the effects on adjacent mucosa, local microbiota, and long-term tissue repair should also be assessed.

Safety considerations are more complex for cell-membrane-coated nanoparticles, exosomes, and other biomimetic platforms. Differences in donor cells, culture conditions, and isolation procedures can alter membrane-protein composition, vesicle cargo, and immunomodulatory effects. In addition to conventional parameters such as particle size, polydispersity index (PDI), and DL, these systems require rigorous control of donor sources, endotoxin and pathogen contamination, potentially proinflammatory components, biodistribution, and clearance pathways [256]. For cell-membrane and extracellular-vesicle systems with inflammation-homing or immunomodulatory functions, the possibility of unanticipated immune activation or amplification of local inflammation in specific pathological settings should also be considered [257,258]. For catheter coatings, ureteral stent coatings, and nanoparticle–local matrix composite systems, safety evaluation should approximate actual conditions of use as closely as possible. Continuous urine washout, fluctuations in pH and ionic strength, protein deposition, environmental changes induced by urease-positive organisms, mechanical friction, and catheter bending can all influence structural integrity, release kinetics, and the risk of particle shedding [53,102,259].

Accordingly, in addition to local mucosal compatibility, studies should incorporate long-term indwelling or repeat-dosing conditions to systematically assess particle or metal-ion release, distribution in major organs, immunotoxicity, degradation products, microbiota-related effects, and safety boundaries after cumulative exposure.

#### 7.2.3 Manufacturing and quality-control barriers

Nanoparticle size, PDI, surface charge, morphology, encapsulation efficiency, DL, release profiles, and surface-protein retention can substantially influence *in vivo* distribution and therapeutic effects [179]. Laboratory-scale nanoformulations may show favorable activity but develop particle-size drift, drug leakage, batch variability, reduced storage stability, or sterilization incompatibility during scale-up [257,259,260]. Quality-control requirements are higher for cell-membrane-coated nanoparticles, exosomes, and nanoparticle-hydrogel composites, which require assessment of membrane-protein retention, vesicle cargo, endotoxin levels, gelation behavior, adhesion strength, mechanical properties, degradation rate, and release stability [261]. Catheter coatings should also retain antibacterial or release performance after sterilization, transport, insertion, bending, and long-term indwelling use [262].

#### 7.2.4 Limited clinical evidence and endpoint validation

Randomized controlled trials of nanotherapies for inflammatory urinary disorders remain limited, and most candidate systems are still at the *in vitro* or animal-model stage [[263], [264], [265]]. Some antimicrobial-coated catheters are clinically used, but their effects on symptomatic CAUTI, antibiotic use, catheter obstruction, re-catheterization, and long-term outcomes remain controversial [52]. For noninfectious inflammation, high-quality clinical data on pain, urinary frequency, quality of life, recurrence, and disease control are even more limited.

Clinical endpoints should be disease specific. For recurrent UTI, key outcomes include culture-confirmed recurrence frequency, time to recurrence, and antibiotic use [266]. For CAUTI, endpoints should include symptomatic infection, catheter patency, encrustation, and obstruction. For IC/BPS, pain, urinary symptoms, patient-reported outcomes, and repeat-treatment needs are important. For CP/CPPS, evaluation can include the NIH Chronic Prostatitis Symptom Index (NIH-CPSI), pain subscale scores, urinary symptoms, and quality of life [267]. Linking material-related measures with patient-relevant outcomes is essential for assessing incremental clinical value.

#### 7.2.5 Functional integration and translational complexity

Multicomponent and multifunctional designs can expand the therapeutic capabilities of nanosystems, but they also increase the complexity of manufacturing, characterization, quality control, and safety evaluation [[268], [269], [270]]. In local delivery systems, the necessity of adding material components, targeting ligands, or responsive modules should be judged in relation to the principal disease barrier, route of administration, and intended therapeutic endpoints [271,272]. For example, intravesical instillation is limited by urine washout and insufficient local exposure, making mucoadhesion and controlled release direct priorities. If additional targeting, imaging, or multidrug modules are added, comparisons with the base carrier or simplified formulations should demonstrate clear pharmacological benefit. For CAUTI coatings, layered designs may improve early anti-adhesive performance, sustained antibacterial activity, and anti-encrustation effects, but their adhesion stability, particle-release risk, and mechanical impact still require verification. For CP/CPPS, local anti-inflammatory or antioxidant delivery will be clinically meaningful only if it also addresses pain sensitization, pelvic-floor dysfunction, or other symptom-related pathology.

Therefore, the selection of functional modules should be based on clearly defined pathological barriers and delivery requirements. Each component should have an interpretable functional role, and comparative studies should define its incremental contribution to local exposure, therapeutic efficacy, safety, or clinically relevant endpoints.

### 7.3 Future directions for nanoparticles in the treatment of inflammatory diseases of the urinary system

In the future, nanoparticles are more likely to optimize existing treatment strategies than to replace standard therapies. Research should prioritize disease-model appropriateness, long-term safety, delivery-route compatibility, manufacturing reproducibility, and patient-relevant outcomes. For UTI and CAUTI, nano-enabled antibacterial systems may complement guideline-based antibiotics, urinary anti-adhesion strategies, catheter management, and recurrence-prevention regimens [16]. For IC/BPS, nanoparticle-assisted intravesical instillation may be combined with behavioral treatment, bladder training, pelvic-floor physical therapy, analgesia, and barrier repair to improve retention, penetration, and inflammation control [219]. For CP/CPPS, prostate-targeted delivery or pelvic-floor-directed treatment may be integrated with α-blockers, anti-inflammatory drugs, neuromodulation, rehabilitation, and psychological intervention [208].

Stimuli-responsive nanosystems remain an important direction [273]. Infectious and inflammatory microenvironments often show pH shifts, elevated ROS, altered enzyme activity, metabolic remodeling, and immune-cell infiltration; pH-, ROS-, enzyme-, or multistimuli-responsive nanocarriers can use these cues to regulate release and reduce normal-tissue exposure [[274], [275], [276], [277]]. In infectious inflammatory disease, they can increase antibiotic release in bacteria-rich regions, deep biofilms, or intracellular reservoirs [278]. In IC/BPS and CP/CPPS, they may target oxidative stress, inflammasome activation, NGF- and TRPV1-related pain pathways, and mucosal injury [279,280]. Photoresponsive systems, including photodynamic therapy and photothermal therapy, also deserve further investigation [281]. Endoscopic instruments, including cystoscopes, ureteroscopes, and optical fibers, can facilitate local photoactivation for recalcitrant biofilms, localized chronic inflammation, and precise lesion intervention [282,283], although light accessibility, penetration depth, and thermal or oxidative safety boundaries must be defined.

Composite local delivery platforms are promising for inflammatory urinary disorders. They combine nanoparticle-mediated drug protection, tissue penetration, target recognition, or stimuli-responsive release with the local retention and sustained-release functions of matrices, adhesive interfaces, or device surfaces, thereby enhancing local exposure, resisting urine washout, and enabling controlled or sequential release [284,285]. Examples include nanoparticles incorporated into thermosensitive or mucoadhesive hydrogels, drug nanocarriers integrated with catheter or stent coatings, and urethral or rectal depot systems. For IC/BPS, mucoadhesive and sustained-release carriers may prolong bladder residence; for CAUTI, catheter surfaces must balance anti-adhesion, antibacterial activity, and anti-encrustation; and for CP/CPPS, rectal or urethral systems should improve mucosal retention and periprostatic delivery. Each functional unit should have a defined role and demonstrable contribution compared with simplified systems.

Biomimetic and naturally derived nanosystems may further expand local delivery strategies [286]. Nanoparticles coated with macrophage, neutrophil, platelet, or erythrocyte membranes can exploit membrane-protein-mediated immune evasion, inflammation targeting, pathogen binding, or cell–cell recognition to enhance local accumulation [241,242]. In CAUTI, recurrent UTI, IC/BPS, and CP/CPPS, immune-cell-membrane-biomimetic nanoparticles may be used for bacterial toxin adsorption, inflammatory cytokine neutralization, immune-cell reprogramming, and local microenvironment modulation [242]. Exosomes and engineered EVs can deliver microRNAs, proteins, anti-inflammatory factors, or repair signals. MSC-derived EVs or EVs from conditioned cells may improve bladder inflammation and urothelial injury through immunomodulation and tissue repair [287,288]. Future work should prioritize source standardization, cargo stability, potency assays, and long-term safety.

Integrating nanotechnology with gene therapy, immunomodulation, and cell therapy may further broaden applications in chronic inflammatory urinary disorders. Lipid nanoparticles, polymeric nanoparticles, exosomes, and cell-membrane-biomimetic platforms can deliver siRNA, microRNA, mRNA, asODNs, or gene-editing systems targeting NF-κB, NLRP [3], NGF, TRPV1, TLR4, IL-17, and related inflammatory, pain, and mucosal-injury pathways. For IC/BPS and CP/CPPS, these strategies may enable more durable suppression of inflammation, restoration of immune balance, and pain control. For recurrent UTI, nanovaccines or mucosal immune-delivery systems may support active immune prevention [145,289,290]. Nanoplatforms may also regulate macrophage polarization, neutrophil recruitment, T-cell responses, or local immune tolerance, shifting treatment toward restoration of immune homeostasis.

Precision-oriented design will be critical to the clinical value of nanotherapy, particularly for heterogeneous disorders such as IC/BPS and CP/CPPS. Individual patients may be driven predominantly by urothelial barrier injury, immune dysregulation, oxidative stress, neural sensitization, pelvic-floor dysfunction, fibrotic remodeling, or microbial abnormalities. Future patient stratification may therefore integrate transcriptomic, proteomic, metabolomic, urinary biomarker, microbiome, and imaging data to identify dominant pathological features and guide nanoparticle selection [291,292]. For IC/BPS, barrier-dominant phenotypes may be preferentially matched with GAG nanostructures, liposomes, or mucoadhesive hydrogels, whereas inflammation/oxidative-stress-dominant phenotypes may benefit from ROS-responsive carriers, nanozymes, cerium oxide, or PDA-based materials. Patients with prominent pain sensitization may instead require local drug or nucleic-acid delivery targeting NGF, TRPV1, or related neuroinflammatory pathways. For CP/CPPS, the UPOINT system may provide a clinically applicable framework for integrating nanotherapy into phenotype-directed management. Organ-specific inflammatory phenotypes may be better suited to prostate-targeted, sustained-release, or inflammation-responsive nanoplatforms, whereas antimicrobial nanodelivery should be considered primarily when an infection-related component is identified. Neurologic/systemic phenotypes may provide opportunities for future pain- or neuroinflammation-targeted nanotherapy, while psychosocial and pelvic-floor tenderness domains should remain primarily addressed through established multimodal interventions. Thus, nanotherapy may be positioned as a phenotype-matched component of individualized treatment rather than a uniform strategy for all patients. Artificial-intelligence-assisted material screening, release prediction, and efficacy modeling may further improve platform selection and support individualized therapeutic strategies (Fig. 9) [293,294].

Overall, the advancement of nanotherapy for inflammatory disorders of the urinary system requires that material selection, disease mechanisms, delivery routes, safety assessment, manufacturability, and clinical endpoints be considered within a unified translational framework. The value of a nanosystem lies not in simple accumulation of functional units, but in its ability to provide verifiable additional benefit for defined delivery barriers and clinical needs. Only when efficacy, safety, and implementation feasibility are established can nanodelivery systems progress from preclinical efficacy to clinical utility.

## CRediT authorship contribution statement

**Wang Wang:** Writing – original draft. **Jiayi Ma:** Writing – original draft. **Changhao Hou:** Writing – original draft. **Jiasheng Chen:** Writing – review & editing, Conceptualization. **Kai Ni:** Writing – review & editing, Supervision, Project administration, Funding acquisition, Conceptualization.

## Ethics approval and consent to participate

Not applicable.

## Consent for publication

Not applicable.

## Fundings

This work was supported by the 10.13039/501100001809National Natural Science Foundation of China [grant number 82103260 to K.N.]; the 10.13039/501100013105Shanghai Rising-Star Program [grant number 22QA1407100 to K.N.]; the Excellent Youth Cultivation Program of Shanghai Sixth People's Hospital [grant number ynyq202204 to K.N.]; the Fundamental Research Funds of Shanghai Sixth People’s Hospital (grant number: X-2490 to K.N.).

## Declaration of competing interest

The authors declare that they have no known competing financial interests or personal relationships that could have appeared to influence the work reported in this paper.

## Data Availability

No data was used for the research described in the article.
